# Sticking Together an Updated Model for Temporary Adhesion

**DOI:** 10.3390/md20060359

**Published:** 2022-05-27

**Authors:** Philip Bertemes, Alexandra L. Grosbusch, Anik Geschwindt, Bob Kauffmann, Willi Salvenmoser, Birte Mertens, Robert Pjeta, Bernhard Egger, Peter Ladurner

**Affiliations:** 1 Institute of Zoology, University of Innsbruck, 6020 Innsbruck, Austria; philip.bertemes@uibk.ac.at (P.B.); alexandra.grosbusch@uibk.ac.at (A.L.G.); anik.geschwindt@student.uibk.ac.at (A.G.); bob.kauffmann@student.uibk.ac.at (B.K.); willi.salvenmoser@uibk.ac.at (W.S.); birte.mertens@i-med.ac.at (B.M.); robert.pjeta@gmail.com (R.P.); bernhard.egger@uibk.ac.at (B.E.); 2 Center of Molecular Biosciences Innsbruck, University of Innsbruck, 6020 Innsbruck, Austria

**Keywords:** Polycladida, non-permanent adhesion, glue, aquatic, duo-gland adhesive system, RNA interference, in situ hybridisation

## Abstract

Non-parasitic flatworms are known to temporarily attach to the substrate by secreting a multicomponent bioadhesive to counteract water movements. However, to date, only species of two higher-level flatworm taxa (Macrostomorpha and Proseriata) have been investigated for their adhesive proteins. Remarkably, the surface-binding protein is not conserved between flatworm taxa. In this study, we sequenced and assembled a draft genome, as well as a transcriptome, and generated a tail-specific positional RNA sequencing dataset of the polyclad *Theama mediterranea*. This led to the identification of 15 candidate genes potentially involved in temporary adhesion. Using in situ hybridisation and RNA interference, we determined their expression and function. Of these 15 genes, 4 are homologues of adhesion-related genes found in other flatworms. With this work, we provide two novel key components on the flatworm temporary adhesion system. First, we identified a Kringle-domain-containing protein (Tmed-krg1), which was expressed exclusively in the anchor cell. This in silico predicted membrane-bound Tmed-krg1 could potentially bind to the cohesive protein, and a knockdown led to a non-adhesive phenotype. Secondly, a secreted tyrosinase (Tmed-tyr1) was identified, which might crosslink the adhesive proteins. Overall, our findings will contribute to the future development of reversible synthetic glues with desirable properties for medical and industrial applications.

## 1. Introduction

Many organisms have developed specialised adhesion systems to attach to a substrate—a phenomenon known as bioadhesion [1,2,3]. Bioadhesion is involved in fundamental behaviours such as locomotion, mating, and feeding. In aquatic animals, three different modes of bioadhesion are known: permanent, transitory, and temporary adhesion [4,5]. For example, larval stages of ascidians secrete a strong, fast-curing, permanent cement to anchor themselves to the substrate [6]. Another prominent example exercising permanent adhesion is the mussel *Mytilus edulis*, which uses a highly specialised structure, known as byssus, consisting of a blend of different proteins to anchor itself to the substrate [7,8]. The main ingredient in mussel adhesion is 3,4-dihydroxyphenyl-L-alanine (L-DOPA), which is an enzymatically modified tyrosine residue in mussel foot proteins [9,10].

In contrast to permanent adhesion, many aquatic animals produce a reversible glue, which obtains adhesive properties within a very short time (e.g., to avoid dislodgement by water currents or waves), but which allows for voluntary and quick detachment (reviewed in [11]). For example, echinoderms, such as sea stars and sea urchins, have evolved so-called tube feet, which contain (among others) adhesive and de-adhesive substance-producing cells. They are currently being investigated for the constituents of these multicomponent glues, as well as the releasing agent(s) [12,13,14,15,16,17,18,19,20]. A total of 16 potentially adhesive proteins were found in the sea urchin *Paracentrotus lividus* [18]. A multitude of different secreted proteins were identified in the sea star *Asterias rubens* [14,17,21,22]. For some of these proteins, a putative involvement in the attachment to the interface was described, whereas other proteins are thought to play a role in forming a structural meshwork between the glue and the animal (cohesive function). In such adhesive systems, no L-DOPA has been described to date.

Animals of the large group of free-living Platyhelminthes (flatworms), also known as “Turbellaria”, have evolved a duo-gland adhesive system, often located on the ventral side of their tail [23]. The duo-gland adhesive system is composed of three different cell types: an adhesive gland cell, a releasing gland cell, and a modified epidermal cell, termed the anchor cell [23]. In the early branching marine macrostomid flatworm *Macrostomum lignano*, two large proteins, namely *Macrostomum lignano* adhesion protein 1 (Mlig-ap1, 5407 amino acids) and *Macrostomum lignano* adhesion protein 2 (Mlig-ap2, 14,794 amino acids), are involved in reversible wet adhesion [24,25,26]. Mlig-ap2 is in contact with the surface, whereas Mlig-ap1 acts as a cohesive protein, which is thought to play a role in connecting Mlig-ap2 to the microvilli of the anchor cell. Two large, low-complexity regions flank Mlig-ap1, whereas Mlig-ap2 has a repetitive core that spans over nearly two-thirds of the whole protein [26]. Recently, the adhesive system of six other macrostomid flatworms inhabiting fresh, brackish, or seawater environments were thoroughly described, and a high similarity of adhesive proteins independent of the aquatic environment was reported [27].

A single species of another free-living flatworm order was thoroughly analysed for its adhesive proteins, namely the proseriate *Minona ileanae* [28]. In contrast to macrostomids, *M. ileanae* secretes a blend of several different proteins to attach to a substrate. In *M. ileanae*, the ortholog of Mlig-ap1 was split into two different proteins, Mile-ap1 and Mile-ap3. The latter consists only of the low-complexity glycine-arginine-lysine-rich repetitive regions (GRK repeats) that flanked ap1 in *M. lignano*, whereas Mile-ap1 is similar, in terms of conserved protein domains, to the core region of Mlig-ap1. In addition to these proteins, the anchor-cell-specific intermediate filament first described in *M. lignano* as macif1 was also present in *M. ileanae* [28,29]. Moreover, three novel flatworm adhesive proteins, Mile-ap4 and Mile-ap5, as well as adhesive organ protein 1 (Mile-ao1), were described [28]. Due to the repetitive nature of adhesive proteins, Mile-ap2 and Mile-ap3 were not completely assembled into single transcripts but into two parts that could not be connected. However, using Oxford Nanopore ultra-long genomic reads, the proximity of both halves was confirmed [28].

In addition to L-DOPA, which was only described in permanent adhesion, it was shown that other post-translational modifications (PTMs) might also play a role in wet adhesion [13,16,22,26,27,30]. The adhesive protein in macrostomids (Mlig-ap2) is glycosylated [26] and was visualised in the adhesive vesicles using peanut agglutinin lectin (a carbohydrate-binding protein) staining [30]. Lectin staining also revealed the presence of ap2 in the footprint (i.e., the material that is left behind upon detachment) of *M. lignano*. Similar results were obtained in other *Macrostomum* species, as well as in the proseriate *M. ileanae* [27,28].

The small sand-dwelling flatworm *Theama mediterranea* (Polycladida) relies solely on its adhesive system to temporarily anchor itself to the substrate [31,32]. In the present work, we describe, for the first time, proteins and mechanisms potentially involved in the adhesive system of polyclads. Our aim was to determine which parts of the adhesive system are conserved throughout the flatworms and which are modified in different groups of flatworms. By comparing the adhesive proteins of different flatworm taxa, we aimed to infer essential features that play a role in flatworm adhesion. Therefore, in the work presented here on *T. mediterranea*, we generated a de novo transcriptome and a positional differential RNA sequencing set specific to the adhesion organ contained in the tail. Using ultra-long genomic reads, we assembled a draft genome, and we were able to tie non-overlapping adhesion protein-coding transcripts to the same genomic region, resulting in longer and more complete potential adhesion-related genes. We showed the expression of these genes using in situ hybridisation. We performed functional analyses of candidate adhesive genes by knocking down selected genes and obtained non-adhesive phenotypes. In addition, we performed lectin staining on whole mounts and footprints to determine whether glycosylation also plays a role in polyclad bioadhesives. Furthermore, we propose a novel mechanism for adhesion mediation in animals between the anchor cell and the secreted adhesive on a substrate.

## 2. Results

### 2.1. Theama mediterranea Adhesive System Morphology

**Figure 1:**
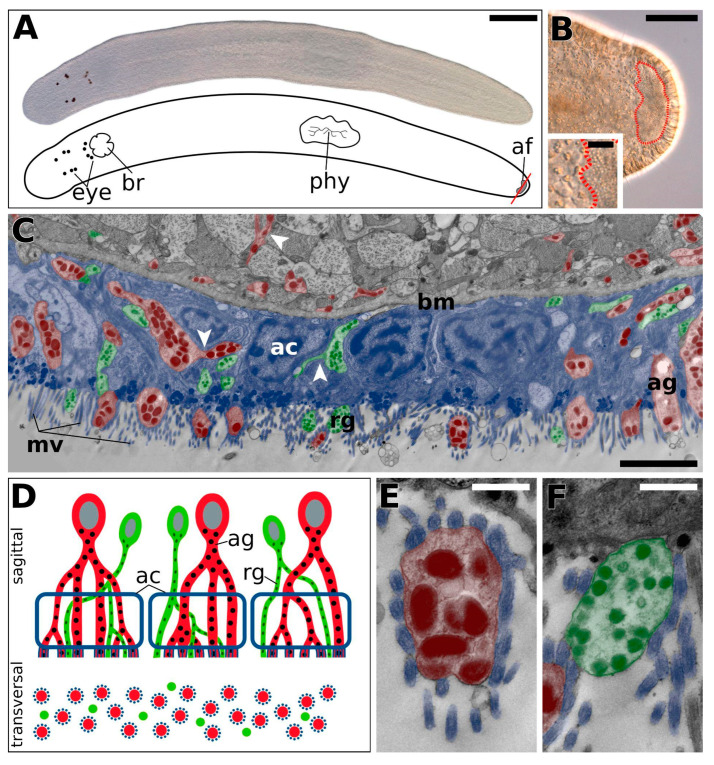
**Morphology of *Theama mediterranea* adhesive organs.** (**A**) Differential interference contrast image of a squeeze-prepared living subadult *T. mediterranea* (**top**) and a schematic drawing (**bottom**). The red transversal line marks the section through the adhesive region shown in (**C**). (**B**) DIC image of the ventral side of the tail, with the adhesive field outlined in red. (**C**) Transmission electron microscope image of a transversal section through the adhesive field, showing the anchor cells (blue), adhesive cells (red) with large electron-dense vesicles, and releasing cells (green) with smaller vesicles. (**D**) Proposed model of the adhesive and releasing gland necks within the adhesive field. (**E**) TEM image of an adhesive gland cell neck in the adhesive field, encased by microvilli. (**F**) TEM image of a releasing gland cell neck in the adhesive field. White arrowheads in (**C**) point to forking gland cell necks. Abbreviations: ac, anchor cell; af, adhesive field; ag, adhesive gland cell; bm, basal matrix; br, brain; eye, eyes; phy, pharynx; mv, microvilli; rg, releasing gland cell. Scale bars: (**A**) 200 µm; (**B**) 50 µm (inset 10 µm); (**C**) 2 µm; (**E**,**F**) 500 nm.

*Theama mediterranea* possess an adhesive field on the tip of the ventral side of the tail, with which they were able to adhere firmly to a substrate (Figure 1A). The adhesive field was located about 30 µm from the tip of the tail and was well distinguishable by interference contrast microscopy (Figure 1B). Transmission electron microscopy revealed that the adhesive field was composed of three cell types, an adhesive gland cell, a releasing gland cell, and a modified epidermal cell called “anchor cell” (Figure 1C). The adhesive gland cells contained many vesicles with an electron-dense, protein-rich inner core and a lucid outer ring (Figure 1E, Figures S1B and S5). Substructures were visible in the inner core of the vesicles (Figure S1B inset). The releasing gland cell contained much smaller vesicles (Figure 1F and Figure S1C). Both gland cell types branched in the anchor cell (Figure 1C,D). Prominent bundles of intermediate filaments were present in the anchor cells (Figure S1D).

### 2.2. Assembly of a T. mediterranea Transcriptome

Four independent batches of animals were collected from sediments from three sampling trips between 2018 and 2021. Total RNA was isolated and subjected to commercial Illumina library preparation. Four individual paired-end libraries of 150 base pairs were sequenced on an Illumina HiSeq 4000 system (Illumina, San Diego, CA, USA) (metrics of individual runs in Table S1). This yielded a total of more than 100 million reads (104,100,328). After error correction and trimming, a total of 86,888,091 reads were used to assemble the first *de novo* transcriptome of *T. mediterranea*.

The Trinity-assembled transcriptome contained a total of 273,517 Trinity ‘genes’ and 591,627 transcripts, with a GC content of 42.09% and an average length of 947.77 base pairs (file deposited at doi:10.5281/zenodo.6470295). In total, 560,724,842 bases were assembled. The annotation pipeline was able to add 94,429 BLASTX hits, 79,111 BLASTP hits, 32,403 predicted transmembrane helix–loop–helix signatures, and 15,043 predicted signal-peptide signatures to the transcripts. BUSCO (in transcriptome mode) reported a transcriptome completeness score of 91.8%. In total, 876 (91.8%) complete BUSCOs were found, with 506 (53.0%) reported as complete and single-copy and 370 (38.8%) reported as complete and duplicated. A total of 25 (2.6%) of the BUSCOs were fragmented, and 53 (5.6%) were missing.

### 2.3. Identification of Tail-Specific Genes by Differential RNA-seq

**Figure 2:**
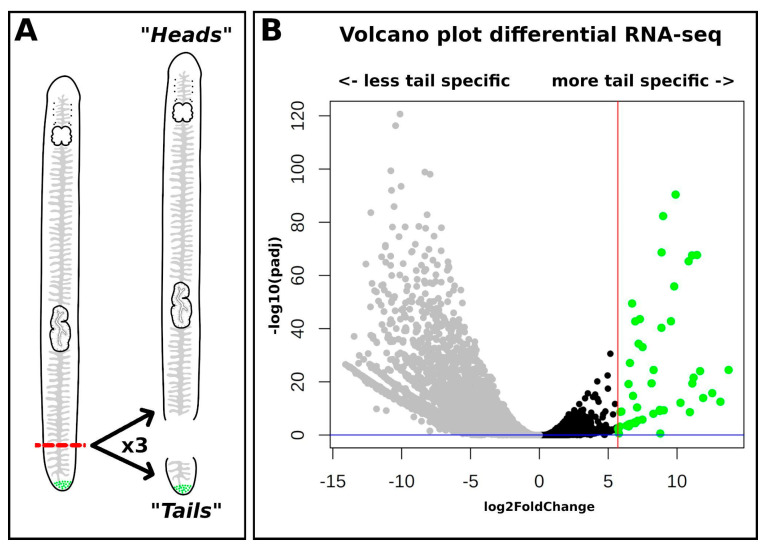
**Volcano plot of the differential RNA-seq dataset.** (**A**) Schematic drawings of the amputation that resulted in the “head” and “tail” pieces for the positional differential RNA-seq. (**B**) Volcano plot of the differentially expressed transcripts. On the *x*-axis, the log_2_-fold change (+ is upregulated in the tail) is plotted against the inverse logarithmic scale of the adjusted *p*-value on the *y*-axis. The blue line represents the cutoff (*p*-value > 0.05); the red line shows the 50× fold-change cut-off (>log_2_(5.64)). In green are the 51 selected candidate transcripts.

The adhesion organs are limited to the posterior part of the tail of *T. mediterranea*. We manually amputated the tail from hundreds of animals and isolated RNA from the anterior and posterior parts of the animals. The anterior parts, called the “heads”, contained the eyes, brain, pharynx, testes, ovaries, and other tissues. The posterior part, the “tails”, contained only the tip of the tail with the adhesive organ (Figure 2A). The positional pieces were then sequenced as single-end 50-base-pair reads on an Illumina system (metrics in Supplementary Table S2). Then, the reads were semi-quantified using a version of the *de novo* transcriptome where duplicated versions of transcripts had been removed, and DESeq2 was used to calculate the differential gene expression (Figure 2B, file “Assay_Tail_Vs_Head_clean.csv” in doi:10.5281/zenodo.6470295). A total of 1479 transcripts were upregulated at least eightfold in the tail, whereas 51 transcripts were upregulated 50-fold in the tail (green dots in Figure 2B). These 51 transcripts were selected for further analysis.

### 2.4. Long-Read Sequencing and Genome Assembly

The large adhesive genes of flatworms identified so far are known to be considerably fragmented in transcriptomes due to the presence of multiple repeat regions and extended sections of low-complexity sequences [26,27,28]. Therefore, we aimed to obtain a genome of *T. mediterranea* to infer the number of repeats and the nature of the low-complexity regions. We performed Oxford Nanopore sequencing using 25 individual sequencing runs, which yielded a total of more than 45.92 gigabases (45,920,210,327) in more than five million reads (5,320,628), with a read length (N50) of 18,653 base pairs. The average quality score was 15.0 (corresponds to an error rate of 3.16%). The longest read was 348,450 base pairs long. The polished draft genome of *T. mediterranea* has a total length of 1,053,348,869 bases in 17,276 contigs, an N50 of 158,223, an L50 of 1923, and a GC content of 40.87%. BUSCO (in genome mode) found 747 (78.3%) complete BUSCOs (single: 694 (72.7%); duplicated: 53 (5.6%)), 86 (9.0%) fragmented BUSCOs, and 121 (12.7%) missing BUSCO genes (out of the 954 BUSCO genes from the metazoa_odb10 data set). A total of 689,902,016 bases (65.50%) were masked as repetitive elements by repeatmasker2.

### 2.5. Genome-Guided Protein Prediction

The braker2 pipeline predicted a total of 37,865 proteins from the draft polished genome. BUSCO (in protein mode) revealed a completeness score of 83.7%. A total of 709 (74.3%) and 90 (9.4%) BUSCOs were found complete and in single or duplicated form, respectively; 47 (4.9%) were fragmented; and 108 (11.4%) BUSCOs were missing.

### 2.6. Selection of Adhesive Protein Candidates

Using BLAST, 50 of the 51 highest exclusively tail-specific expressed transcripts were found on 25 different contigs on the genome, whereas one did not match to a genomic contig. We observed that a few transcripts mapped next to each other on the same contig, meaning that they most likely belong to a single large gene, which probably was not completely assembled in the transcriptome. Overall, eight genes comprised multiple transcripts (one gene comprised 11 transcripts, one gene comprised five transcripts, one gene comprised four transcripts, three genes comprised three transcripts, and two genes comprised two transcripts) (Supplementary Figure S2, files “Transcripts_on_genomic_contigs.ods” and “Shortlist_complete.xlsx” in doi:10.5281/zenodo.6470295), whereas 17 transcripts had no matching partner and were found on individual contigs. Because we only selected highly expressed genes (calculated base mean value higher than 150 in the diff-RNA-seq data set, “Assay_Tail_vs_Head_clean.csv” in doi:10.5281/zenodo.6470295), 16 transcripts were removed from the selection, leaving us with a total of 35 transcripts on 15 different genes.

**Figure 3:**
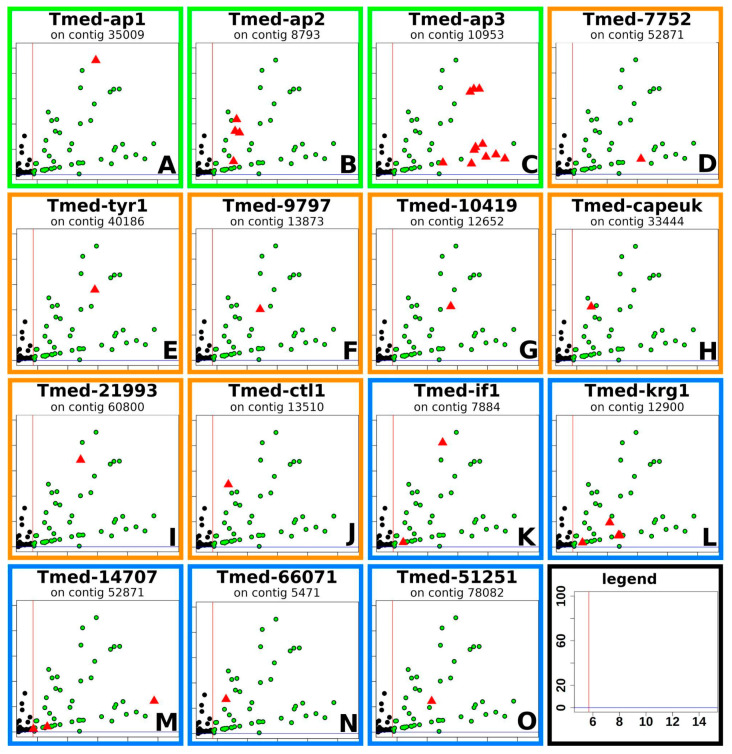
**Candidate selection based on the differential RNA-seq dataset.** The plots show the log_2_-fold change on the *x*-axis plotted against the negative log_10_ of the adjusted *p*-value (both values are calculated by DESeq2). Visualised in green are all transcripts that are (1) upregulated by at least 50-fold (right of red line) and (2) adjusted *p*-value of less than 0.05 (above the blue line is highly significant). Note that some genes are split into multiple transcripts due to the repetitive nature of adhesive proteins (e.g., Tmed-ap2 consists of four transcripts, and Tmed-ap3 consists of 11 transcripts). The colours of the borders around each panel ((**A**–**C**), green; (**D**–**J**), orange; (**K**–**O**), blue) correspond to the expression in the tissue of the animal (green, adhesive cell; orange, adhesion-related cell; blue, anchor cell; see also Figure 5).

In the end, we could thus select 15 highly expressed (Table 1), tail specific and putative adhesion-related genes, namely adhesion proteins 1–3 (Tmed-ap1, Tmed-ap2, Tmed-ap3), Tyrosinase-like 1 (Tmed-tyr1), intermediate filament-like (Tmed-if1), Kringle-like (Tmed-krg1), cysteine-rich secretory protein-like (Tmed-capeuk), c-type lectin-like (Tmed-ctl1), as well as seven transcripts without any known protein domains: Tmed-7752, Tmed-9797, Tmed-10419, Tmed-14707, Tmed-21993, Tmed-51251, and Tmed-66071. Their respective positions in the differential RNA-seq analysis can be found in Figure 3.

**Figure 4:**
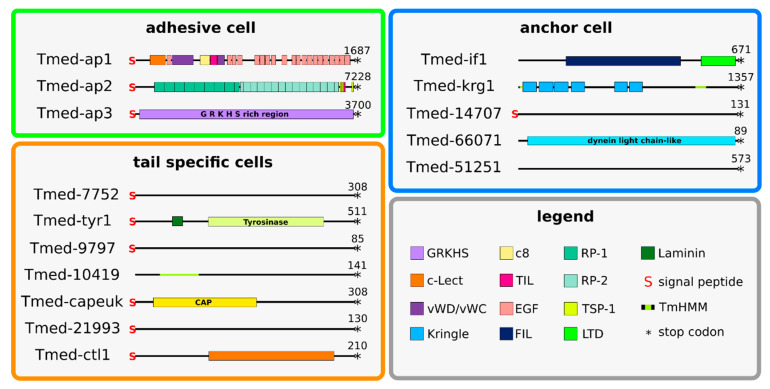
**Conserved protein domains of *T. mediterranea* tail-specific genes.** Tmed-ap1, Tmed-ap2, and Tmed-ap3 are the three genes that are found in the adhesive cell of *T. mediterranea* (green box). Tmed-7752, Tmed-tyr1, Tmed-9797, Tmed-10419, Tmed-capeuk, Tmed-21993, and Tmed-ctl1 are tail-specific genes and are potentially involved in the adhesive system (orange box). Tmed-if1, Tmed-krg1, Tmed-14707, Tmed-66071, and Tmed-51251 are anchor-cell-specific genes (blue box). Abbreviations: c8, domain of eight conserved cysteines; CAP, cysteine-rich secretory protein; c-Lect, c-type lectin domain; EGF, calcium-binding epidermal growth factor-like domain; FIL, intermediate filament protein domain; LTD, lamin tail domain; RP-1, repeat motif 1; RP-2, repeat motif 2; TIL, trypsin inhibitor-like domain; TmHMM, transmembrane domain; TSP-1, thrombospondin domain; vWD, von Willebrand type D domain; vWC, von Willebrand type C domain.

**Figure 5:**
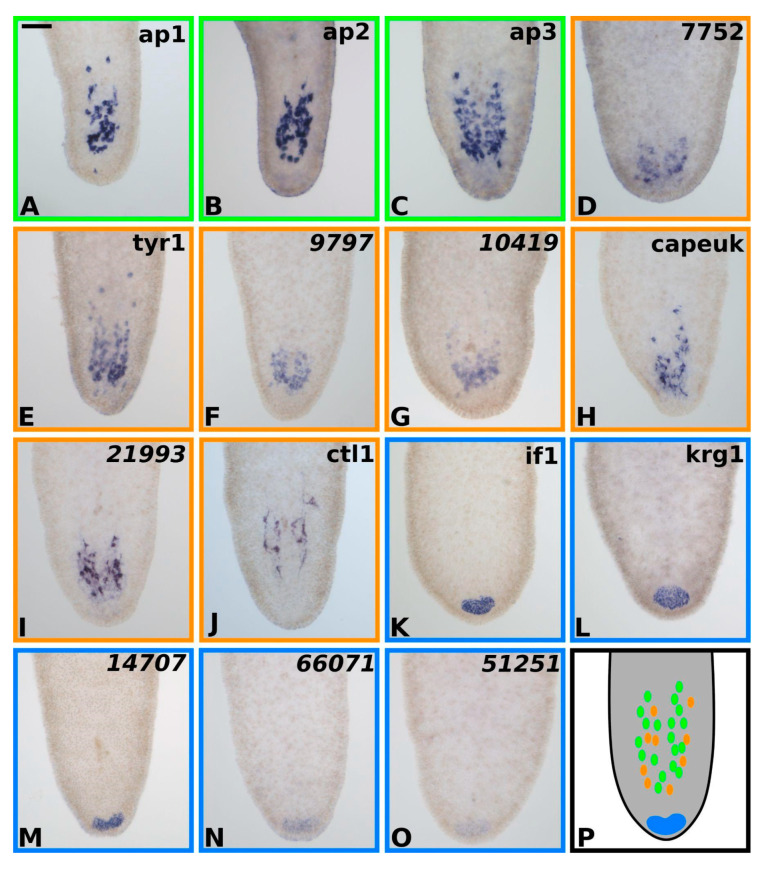
**In situ hybridisations of tail-specific genes.** Expression of all 15 candidate genes visualised using in situ hybridisation. Three different patterns became apparent: adhesive-organ-specific ((**A**–**C**) encased in green), tail-specific ((**D**–**J**) encased in orange), and anchor-cell-specific ((**K**–**O**) encased in blue). (**P**) Schematic drawing of the distribution of the ISH patterns. Scale bar: 50 µm.

These selected genes (Figure 3 and Figure 4) were classified into cell types according to their expression in the tail (Figure 5). Based on the homology of Tmed-ap1, Tmed-ap2, and Tmed-ap3 to previously published adhesion-related genes from other flatworm groups [26,28], it is highly likely that those genes are present in the adhesive cells (Figure 3, Figure 4 and Figure 5; green box). Five transcripts had an expression in the anchor cell: intermediate filament (Tmed-if1) and Kringle-like (Tmed-krg1), as well as transcripts 14,707, 66,071, and 51,251 (Figure 3, Figure 4 and Figure 5; blue box). Another seven transcripts were expressed in the tail but could not be allocated to a certain cell type, comprising a tyrosinase-like (Tmed-tyr1), a cysteine-rich secretory domain containing protein (Tmed-capeuk), and a c-type lectin-like protein (Tmed-ctl1), as well as the transcripts 7752, 9797, 10,419, and 21,993 (Figure 3, Figure 4 and Figure 5; orange box).

#### 2.6.1. Adhesion Proteins

The three adhesive proteins, Tmed-ap1, Tmed-ap2, and Tmed-ap3, were named after their homology to proteins in the flatworms *Minona ileanae* and *Macrostomum lignano* (Figure 4, green box). Adhesion protein 1 (Tmed-ap1) is a 1687-amino-acid-long protein with several conserved domains; at the N-terminal end, a signal peptide was predicted, followed by a C-type lectin domain (c-Lect), a calcium-binding epidermal growth factor-like domain (EGF), a von Willebrand type D domain (vWD), a domain of eight conserved cysteines (C8), a thrombospondin-like domain (TIL), a von Willebrand type C domain (vWC), and 19 EGF domains. Adhesion protein 2 (Tmed-ap2) is 7228 amino acids long and contains a signal peptide in its N-terminal end. In its central region, two highly repetitive regions were found, which contain 8 (+1 partial) repetitions of a 330-amino-acid-long stretch and 12 (+2 partial) repetitions of a 230-amino-acid-long stretch. The C-terminal end contains several known conserved domains, such as three thrombospondin- and one trypsin inhibitor-like domain. Tmed-ap2 contains 1625 lysine amino acid residues, which constitute 22.5% of the whole protein. In addition, a total of 819 (11.33%) amino acids of ap2 have predicted O-glycosylation motifs. Adhesion protein 3 (Tmed-ap3) was predicted as a 3700-amino-acid-long protein; a signal peptide can be found at its N-terminal end. The largest portion of this protein is a low-complexity region, which contains a “GRKHS” motif mainly composed of five amino acids: lysine (1051 aa), arginine (880 aa), glycine (581 aa), histidine (444 aa), and serine (288 aa). Those five amino acids constitute 87.67% of Tmed-ap3.

#### 2.6.2. Anchor-Cell-Specific Proteins

Five genes were expressed exclusively in the anchor cells of the tail (Figure 4, blue box). One of them, *Theama mediterranea* intermediate filament-like protein (Tmed-if1), consisted of 671 amino acids and contained two conserved protein domains, one known as intermediate filament domain; the other, known as the lamin tail domain, was located at the C-terminal end of the protein. In addition, we found a highly expressed anchor-cell-specific protein with considerable size (1367 aa). It was predicted to have a large extracellular region (aa positions 29-1091) flanked by intracellular regions. The extracellular region contains six concurrent lysine-binding Kringle domains. Tmed-14707 (131 aa long) contained a signal peptide but no conserved domains. Tmed-66071 (81 aa long) contained a dynein light-chain-like protein domain. Tmed-51251 was 573 amino acids long, but it contained neither a conserved domain nor a signal peptide or a transmembrane (TMM) region.

#### 2.6.3. Tail-Specific Proteins

This group (Figure 4, orange box) consists of seven different proteins coded by genes with expression exclusively in the tail (Figure 5, orange box). Six of these seven proteins (Tmed-7752, Tmed-tyr1, Tmed-9797, Tmed-Tmed-capeuk, Tmed-21993, and Tmed-ctl1) contained a signal peptide. One of them (Tmed-10419) did not contain a signal peptide but a TMM region. Only three proteins contained conserved protein domains: Tmed-tyr1 is a 511-amino-acid-long protein that contains a signal peptide, a laminin domain and a tyrosinase domain; Tmed-capeuk is 308 amino acids long and contains a CAP region, which is a conserved cysteine-rich secretory protein-like domain; and Tmed-ctl1 is a 210-amino-acid-long protein that contains a C-type lectin domain in its N-terminal end. The other four proteins, Tmed-7752 (308 aa), Tmed-9797 (85 aa), Tmed-10419 (141 aa), and Tmed-21993 (130 aa), did not contain any conserved domains.

### 2.7. Localisation of Candidate Genes by In Situ Hybridisation

Using digoxigenin-labelled RNA probes targeting each of the different candidate genes, we were able to confirm their expression in the tail of the animals. The cell bodies of adhesive cells in flatworms are sunk far into the tail of the animal [4,23,28]. The cell bodies in *T. mediterranea* were observed to easily reach up to 100 µm towards the anterior from the tip of the tail (data not shown). Combining this knowledge from *T. mediterranea* with published data on where to expect adhesive candidate genes from other flatworms, we are confident to report that the expression of the genes Tmed-ap1, Tmed-ap2, and Tmed-ap3 are also localised exclusively in the adhesive cells (Figure 5A–C). The gene Tmed-if1 is a marker of anchor cells in other flatworm species, and it is also exclusively expressed in the adhesive field of *T. mediterranea* (Figure 5K). The genes Tmed-krg1, Tmed-14707, Tmed-66071, and Tmed-51251 showed the same expression pattern as the anchor cell-specific marker Tmed-if1 (Figure 5L–O). We therefore assigned these genes as exclusive to the anchor cell. For the genes Tmed-tyr1, Tmed-capeuk, and Tmed-ctl1, as well as the transcripts Tmed-7752, Tmed-9797, Tmed-10419, and Tmed-21993, we detected an exclusive localisation in the tail of the animals, similar to the pattern observed in the adhesive gland cell (Figure 5D–J).

### 2.8. Functional Analysis of Candidate Genes by RNA Interference

We synthesised double-stranded RNA (dsRNA) to individually knock down the genes Tmed-ap1, Tmed-ap2, Tmed-ap3, Tmed-7752, Tmed-tyr1, Tmed-if1, Tmed-krg1, Tmed-ctl1, Tmed-21993, Tmed-capeuk, and Tmed-14707 on freshly tail-amputated adult animals. By amputating the tails, all adhesion-related cells were removed from the animals. A negative control was performed using an off-target gene (luciferase), and in the double-negative control, no dsRNA was added to the regenerating animals. Animals were checked every day for adherence. After 11 days, control animals (non-treated and luciferase off-target) regained the ability to temporarily adhere to the substrate.

On the twelfth day of the knockdown experiment, three independent researchers checked three individuals in each group in a double-blind study. We noted non-adhesive phenotypes in Tmed-ap2, Tmed-krg1, Tmed-if1, and Tmed-7752 knockdown animals. No phenotype was detected for the other genes. However, we had no measure to determine whether the RNA interference experiment was unsuccessful, whether the genes were not directly involved, or were not substantial in temporary adhesion.

### 2.9. Glycosylation Detection in Whole Mounts and Footprints by Lectin Staining

Adhesion protein 2 is predicted (netOGlyc v4.0.0.13) to be a highly glycosylated protein with 819 potential O-glycosylation sites. It has been shown in other flatworms that lectins, which are carbohydrate-binding proteins, can be used to stain different sugar moieties. Peanut agglutinin (PNA) is known to bind with high specificity to the sugar galactosyl (β-1,3) N-acetylgalactosamine [6,22,30]. The adhesive field of *T. mediterranea* was stained by the lectin peanut agglutinin (PNA) in whole-mount stainings, and, correspondingly, (Figure S4A,B between arrowheads) footprints left behind by the animals after detachment were also stained with the lectin PNA. This staining revealed a distinct PNA-positive footprint on the glass slide (Figure S4C), as well as mucus pathways (Figure S4D).

### 2.10. No L-DOPA Was Found in the Footprints

We identified a tyrosinase expressed in the tail of *T. mediterranea*. Therefore, we considered that L-DOPA might be present in the footprints. However, we could not confirm the presence of L-DOPA residues in *T. mediterranea* footprints. In addition to antibody staining, nitroblue tetrazolium staining, as described in Zeng et al. [6], also did not result in any staining of the footprint.

## 3. Discussion

### 3.1. Adhesive System Morphology

Flatworms make use of a temporary adhesive system to attach themselves to substrates during heavy tidal action in order to avoid dislodgment from the sediment. However, this quick-setting firm bond needs to be reversible to accommodate their non-sessile lifestyle. Flatworms have evolved a specialised organ that comprises a cell that produces an adhesive substance (adhesive cell), a cell that produces a de-adhesive agent (releasing cell), and a cell that is responsible for handling the forces between the interface and the animal (anchor cell). The cell bodies of the gland cells are often deeply sunk into the tail-plate of the animal, with long gland cell necks, which penetrate a single or multiple anchor cell/s to apically discharge the glue-containing vesicles towards the ventral side of the animal [23]. A common feature among most free-living flatworms is the formation of adhesive papillae, which consists of a microvilli collar formed by the anchor cell encasing the adhesive gland cell neck [23]. One particularity in the early-branching flatworm taxon Macrostomorpha is that adhesive and releasing glands share a microvilli collar [23,24]. In *Macrostomum*, one adhesive cell and one releasing cell will always produce a single adhesive papilla [27], whereas in other flatworm groups, such as Proseriata, Polycladida, Rhabdocoela, and Tricladida, both the adhesive and releasing gland cell necks branch multiple times [23]. In these flatworm taxa, it was reported that the releasing gland cell necks emerge individually from the anchor cell, that they are interspersed between the adhesive papillae, and that they do not have a microvillus collar [23]. *T. mediterranea* exhibited the latter described arrangement; the position of the adhesive organ was constrained to a single adhesive field at the tip of its tail, gland necks branched prior to inserting into the anchor cell, and only the adhesive gland cell necks were encased by the microvillus collar, thus producing the adhesive papilla, whereas the releasing gland cells emerged individually between these papillae. The general organisation of the adhesive organs is very similar to that found in the well described proseriate *Minona ileanae* and its congeners [23,28].

### 3.2. Adhesive Vesicles Are Compartmented

It was recently shown that *M. lignano* secretes two large proteins, Mlig-ap1 and Mlig-ap2, which suffice to adhere to substrates in wet conditions [26]. In addition, this simple two-component glue works under the influence broad range of different external factors, including salinity, pH, and temperature [26]. Both adhesive proteins were located in a single vesicle type, which can be found in the adhesive gland cell of *M. lignano*. These vesicles were shown to have an electron-lucid outer ring and an electron-dense inner core in transmission electronic images [26,29]. In RNA knockdown experiments, it was found that the adhesive protein Mlig-ap2 corresponds to the outer rim, and the cohesive protein Mlig-ap1 represents the inner core of the vesicle [26]. This same vesicle content pattern was also observed in six other *Macrostomum* species, although they occur in different environments (freshwater, brackish water, and sea water), as well as in a proseriate flatworm [27,28]. In *T. mediterranea*, a similar arrangement was identified, with vesicles with a bright ring around a dark inner core (Figure S1B, inset), suggesting a similar protein distribution in the adhesive vesicles as in other flatworms.

### 3.3. Segregation of the Cohesive Protein into Two Different Proteins

Adhesive proteins in aquatic animals often share a similar set of known protein domains, such as a von Willebrand factor type D domain, a C-type lectin domain, a conserved domain of eight cysteine residues, trypsin-inhibitor domains, and a multitude of calcium-binding epidermal growth factor-like binding domains [11]. This combination is found in many adhesion-related proteins across different species, such as echinoderms, molluscs, and flatworms [17,18,27,28,33,34]. In the flatworm *M. lignano*, these protein domains can be found condensed in a central core of the cohesive protein Mlig-ap1 [26]. This core region is flanked at its N- and C-terminal ends by large stretches of a highly repetitive, low-complexity region containing mostly the amino acid residues glycine (G), arginine (R), and lysine (K). A similarity between the proseriate *M. ileanae* and the polyclad *T. mediterranea* (analysed herein) is that these GRK flanks are no longer present on ap1 but as an independent protein, ap3. In *M. ileanae*, ap3 was not completely assembled into a single transcript but was split into ap3a and ap3b due to the limitations of short-read assemblers, such as Trinity, to correctly assemble large repetitive genes. However, in *T. mediterranea*, we were not able to obtain the full-length transcript of Tmed-ap3 from the transcriptome alone, but we were able to reconstruct it in combination with the new genomic resources.

### 3.4. Adhesive Cocktail in Polyclads Rather than Two Components

Next to ap3, which was not found in macrostomids but appeared in proseriates and polyclads, several other adhesive candidate genes were described in the proseriate *M. ileanae*. In addition to *Mile-ap1*, *-ap2*, and *-ap3*, the other new candidate genes (with no homologues in *T. mediterranea*) involved in *M. ileanae* adhesion included *Mile-ap4*, *Mile-ap5*, and *M. ileanae* adhesion organ protein 1 (*Mile-ao1*) [28]. An RNAi knockdown of these genes resulted in an aberrant adhesive vesicle structure and impeded attachment capacities of the worm (except for *Mile-ao1*, where no phenotype was observed). Regarding the complexity of adhesive proteins in *T. mediterranea*, we hypothesise that the contents of the vesicles is not limited to only two components (ap1 and ap2) as in macrostomids but also involves a mixture of the other proteins discovered in the present study.

### 3.5. The Role of Tyrosinase and Post-Transcriptional Modifications in Adhesion

Several post-translational modifications (PTM) have been described in adhesive proteins across different species. Common PTMs in aquatic adhesion are glycosylations [6,16,22,30,35,36,37], serine phosphorylations [38,39], sulfation [40], and the modification of tyrosine, which leads to L-DOPA in mussels [8]. Although extensive knowledge has been generated about L-DOPA, to date, it has been described only in the cements of permanently adhering mussels [8] and the glues of the sandcastle worm *Phragmatopoma californica* [41]. However, the presence of L-DOPA has also recently been suggested in the ascidian *Ciona intestinalis* [6]. Despite extensive trials, we were unable to detect the presence of L-DOPA in the adhesive footprint of *T. mediterranea*. However, the gene coding for the enzyme responsible for oxidising the amino acid tyrosine to L-DOPA, tyrosinase, was found to be present in high abundance with high specificity for the adhesive gland area in *T. mediterranea* (Figure 3E and Figure 5E). The protein Tmed-tyr1 contains a signal peptide (Figure 4), suggesting that it is secreted and that potential reactions happen in the extracellular space. In a first reaction, tyrosinase transforms the amino acid tyrosine to L-DOPA, which is a strong adherent under the right conditions. A second role of tyrosinase is to further oxidise L-DOPA to an activated quinone, which can then crosslink to other activated quinones, forming a network [42,43]. However, if L-DOPA is oxidised to its dopaquinone form, it loses its adhesive properties [9,44]. This oxidation can also happen spontaneously, and mussels need to carefully lower the pH in the adhesive plaque in order to prevent such oxidation [44]. It is rather unlikely that the adhesive papillae in flatworms create a local low-pH environment; therefore, we hypothesise that the first function of tyrosinase (hydroxylation of tyrosine to L-DOPA) is not its main role in flatworm adhesion. In fact, it was shown that tyrosinase 1 in *P. californica* also has a signal peptide, and the white adhesive slowly changes colour during curing, suggesting that the glue undergoes an enzymatic reaction (cross linking) [45]. Improving knowledge on tyrosinase activity was recently utilised to produce fast-curing bio-inspired hydrogels [46,47]. Tmed-tyr1 is therefore an addition to the field of biomimetic glue production.

### 3.6. Genomic Resources Are Essential to Resolve Large Proteins

Adhesive proteins are known to be very large. For example, the sea star adhesive proteins sfp1 and Arub-10 are 3853 and 3716 amino acids long, respectively [15,17]. In flatworms, the adhesive proteins in *M. lignano*, Mlig-ap1, and Mlig-ap2 are 5407 and 14,794 amino acids long, respectively [26]. In *M. ileanae*, the exact length of Mile-ap2 could not be completely resolved [28]. However, by using single ultra-long genomic reads obtained by Oxford Nanopore sequencing, an exon of at least 15,000 base pairs was identified, implying a Mile-ap2 protein of at least 5000 amino acid residues. *T. mediterranea* conforms to tendency to have large adhesive proteins, with Tmed-ap2 being 7228 amino acids long. However, this could not be resolved using transcriptomic resources alone. Tmed-ap2 is a hybrid between the genome-based protein prediction and RNA-seq data, which could only be inferred by sequencing and assembling the first high-quality draft genome of a polyclad flatworm. Likewise, the adhesive protein Tmed-ap3, which contains a highly repetitive GRKHS motif and therefore did not assemble properly in the transcriptome, was fragmented into eleven different transcripts that were reconstituted using the genome data. Therefore, the use of a well-annotated genome in a genome browser (e.g., jbrowse) seems indispensable in working with such large genes. These draft genomic assemblies are therefore a powerful resource to exploit.

### 3.7. Updated Model for Temporary Adhesion

The current flatworm adhesion model relies on the idea that an adhesive (ap2) attaches to the substrate, and a cohesive (ap1) mediates the contact between the adhesive and the glycocalyx covering the microvilli of the anchor cell [26]. A suggested unknown negatively charged substance secreted by the releasing vesicles interacts with the positively charged Mlig-ap1 and increasingly supersedes the connection to the microvilli glycocalyx. It was further shown that with the use of the negatively charged anti-blood coagulation agent heparin, the animals lost their ability to attach; we hypothesised that this molecule masks the positively charged residues in the cohesive protein.

**Figure 6:**
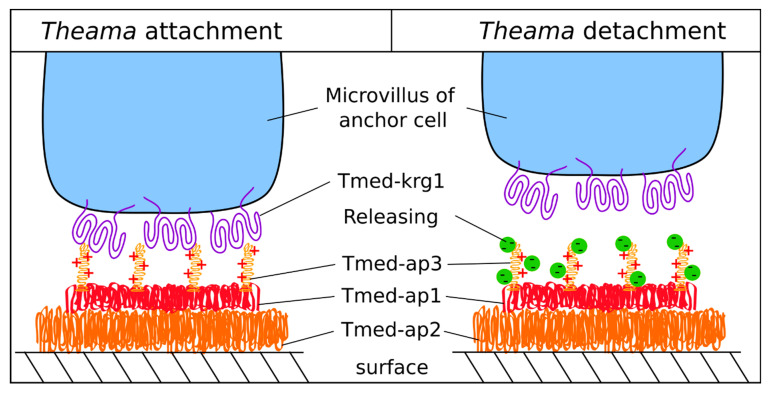
**Schematic of a potential reversible adhesive system in *T. mediterranea*.** This is an oversimplified model, which might not include all involved proteins. **Attachment**: Tmed-ap1, Tmed-ap2 (with predicted o-glycosylation sites), and Tmed-ap3 are secreted and possibly subsequently crosslinked by Tmed-tyr1. The positively charged lysine residues of Tmed-ap3 interact with the Kringle-domain-containing microvilli-membrane-bound Tmed-krg1. **Detachment**: A possibly negatively charged releasing molecule is secreted from the releasing gland cell (not shown) and interferes with Tmed-ap3 binding to Tmed-krg1, additionally masking the positively charged Tmed-ap3-lysines, thus effectively supersedingTmed-krg1 from Tmed-ap3.

In this work, we discovered the anchor cell specific protein Tmed-krg1 (Figure 5L), which contains a large extracellular domain. The parts that were predicted to be extracellular are multiple concurrent Kringle domains (Figure 4). Kringle protein domains are known to take part in blood clotting and can be found in many different proteins that are responsible for coagulation [48]. They have a well-defined structure consisting of three loops and disulphide bridges and create a pocket that is able to bind the amino acid lysine [49,50]. It was shown that the lysine-binding pocket of Kringle-domains also binds heparin and other piperazines [51,52]. Because the cohesive in Tmed-ap3 contains many lysine residues and Tmed-krg1 contains six Kringle domains, we hypothesise that the interaction between cohesives and animals is actually mediated through this particular protein (Tmed-krg1) that is membrane-bound in the anchor cell (Figure 6). To release from a surface, the animal secretes a negatively charged molecule. This molecule is proposed to interact with the positively charged cohesive protein Tmed-ap3, thus masking and outcompeting the lysines that were bound by the Kringle domains, eventually leading to the disintegration of the bond between animal and cohesive protein. It seems that this mechanism is not a peculiarity of *T. mediterranea* but could also be present in other flatworm groups. We found homologues of Tmed-krg1 in the macrostomid *M. lignano*, as well as in the proseriate *M. ileanae*. From the published differential RNA-seq data of these two species, we found the transcript Mii7848_c1_g1_i1 to be upregulated about eightfold in *M. ileanae*, as well as transcript RNA815_8153, which is highly specific for the tail of *M. lignano* [28,53,54]. In *M. lignano*, the published in situ hybridisation also revealed its expression in the anchor cell (Supplementary Figure 3T in Lengerer et al. [54]). However, we did not perform any experiments to localise the Tmed-krg1 protein to the microvilli of the anchor cell. This hypothesis needs to be tested in future experiments.

With this work, we provide a high-quality transcriptome and draft annotated genome of the polyclad *Theama mediterranea*. In addition to this, we hereby provide a broad molecular toolbox including lectin stainings, in situ hybridisation in adult polyclads, and RNA interference-mediated gene knockdown. Further including the cytochemical staining protocols for stem cells and the nervous systems that have already been published for *T. mediterranea* [31] and combined with the high availability of this animal in the field, we propose *T. mediterranea* as a highly valuable candidate for a polyclad flatworm research model.

## 4. Materials and Methods

### 4.1. Sampling and Animal Maintenance

*Theama mediterranea* was sampled from sand collected in Rovinj, Croatia (45.1180406 N, 13.616976 E). At low tide, the upper three to five centimetres of sand were collected using a flat shovel on 16 April 2018, 4 July 2020, and 18 October 2021. The substrate was incubated for 10 min in a 1:1 mixture of 35‰ artificial salt water (ASW, hw^®^-Marinemix professional, Wiegandt, Krefeld, Germany) and 7.14% MgCl_2_ × 6 H_2_O (Carl Roth, Karlsruhe, Germany) in a 2 L bottle with occasional strong agitation (rocking and turning of the bottle). After a final strong agitation, the liquid was immediately poured through a 60 µm mesh. The mesh was rinsed in ASW into a plastic Petri dish, following the manual selection of *T. mediterranea* under a stereo microscope. The collected *T. mediterranea* were kept in a glass Petri dish at 35‰ ASW at 15 °C in darkness without feeding for up to seven months. ASW was changed every 2–4 weeks, but animals were never transferred into a new dish.

### 4.2. Amputation (for Differential RNA-seq)

More than 1200 adult animals were manually amputated under a stereomicroscope using a razor blade. This resulted in the anterior part, termed the “head”, containing, e.g., tissues of the head (eyes and brain), the male and female gonads, and the pharynx, as well as the posterior part, termed the “tail”, which consists mainly of the adhesive organ and tail-specific tissues (see Figure 2A for amputation level). We collected 150, 70, and 70 anterior pieces without the tail (=“heads”) and 400, 450, and 313 posterior pieces comprising only the tail (=”tails”). The RNA of the three biological replicates for “head” and “tail” was extracted individually (described below) and sequenced using single-end 50-base-pair Illumina reads.

### 4.3. RNA Extraction

Adult *T. mediterranea* were selected and transferred several times into new dishes with fresh ASW over the course of several hours in order to eliminate any possible contaminants prior to RNA extraction. Four biological replicates, each with 70 animals, were transferred into 1.5 mL Eppendorf tubes and quickly spun on a bench centrifuge, and the medium was completely removed. A volume of 500 µL TRI Reagent^®^ (Sigma-Aldrich, St. Louis, MO, USA) was added to the tubes, and animals were dissolved by pipetting the liquid with the animals 30–50 times up and down. In addition, the tissue was further homogenised using a Precellys Evolution homogenizer (Bertin Instruments, Montigny-le-Bretonneux, France) with 1.4 mm ceramic beads (2 × 20 s at 5000 rpm with a 20 s pause in between). Then, another 500 µL of TRI Reagent^®^ was added prior to adding 200 µL isopropanol. The mixture was pulse-vortexed for 15 s and incubated for 15 min at room temperature until two phases became visible. Then, they were centrifuged for 20 min at 12.000 g at 4 °C in a precooled centrifuge. Then, the upper liquid phase was transferred into a fresh 1.5 mL Eppendorf tube, and 500 µL isopropanol (Sigma-Aldrich, St. Louis, MO, USA) was added. The tubes were inverted a few times and then incubated at room temperature for 10 min prior to centrifugation for 10 min at 12.000× *g* at 4 °C. Next, the supernatant was completely removed, and the pellet was washed with 1 mL precooled 75% EtOH to ensure that the pellet was dislodged from the bottom of the tube. A subsequent centrifugation step was performed for 5 min at 7500× *g* at 4 °C. Then, the liquid was completely removed, and the pellet was air-dried for approximately 10 min. Finally, the RNA pellet was resuspended in 20 µL UltraPure DNase/RNase-free distilled water (Invitrogen, Waltham, MA, USA). Concentration and purity were measured using a Nanodrop 2000 system (Thermo Fisher Scientific, Waltham, MA, USA), and RNA integrity was verified on a 1% agarose gel in 0.1 × TBE buffer. The RNA was finally stored at −80 °C.

### 4.4. Transcriptome Assembly

Four biological replicates of total RNA (each prepared from 70 animals) were sent for Illumina library preparation and subsequent sequencing at the Duke Center for Genomic and Computational Biology (Durham, NC, USA). The 150 base-pair reads for each replicate were corrected using rcorrector (commit ce5d06b) [55], sanity-checked with TranscriptomeAssemblyTools (commit e2df226, script FilterUncorrectabledPEfastq.py), and finally trimmed with TrimGalore v0.6.4_dev using the flags ‘–paired –retain_unpaired –phred33 –length 36 -q 5 –stringency 1 -e 0.1’ [56]. Then, the reads were assembled using Trinity v2.10.0 with the flags ‘–seqType fq –max_memory 250G –CPU 63 –no_salmon –SS_lib_type RF’ [57].

### 4.5. High-Molecular-Weight Genomic DNA Extraction and Library Preparation

High-molecular-weight genomic DNA (hmw gDNA) was extracted from four biological replicates, each with 125 adult *T. mediterranea* using a Nanobind Tissue Big DNA Kit (Circulomics, Baltimore, MD, USA). Different combinations of pre- and post-treatment of the animals and extracted DNA were performed during the isolation process. Animals were incubated for 10 min in either 50 mg or 100 mg N-acetyl-L-cysteine (Sigma-Aldrich, St. Louis, MO, USA) in 10 mL 35‰ ASW (NAC mucus stripping solution) (see Section 4.14. for recipe) prior to incubation in CT buffer provided with the kit. In addition, one extraction was performed without NAC treatment prior to hmw gDNA isolation. For one extraction, we omitted the buffer CT step. After isolation, we used three different versions of the short-read eliminator kit (XS, normal, XL; Circulomics, Baltimore, MD, USA). The DNA sequencing libraries were prepared with the Nanopore LSK-109 chemicals (Oxford Nanopore Technologies, Oxford, UK). Final library concentration was measured with a Qubit 4 fluorometer (ThermoFisher Scientific, Waltham, MA, USA) and diluted in elution buffer according to the manufacturer’s protocol. The whole process resulted in nine sequencing libraries with a total of 25 individual runs on four Oxford Nanopore Technologies R9.4.1 flow cells (Table 2).

### 4.6. Next-Generation Sequencing

Ultra-long hmw gDNA sequencing was performed using a MinION device (Oxford Nanopore Technologies, Oxford, UK). Base calling was performed using ONT guppy v5.0.15 invoking Nvidia V100 Tesla graphics cards (Santa Clara, CA, USA) at the high-performance computing cluster ‘leo4.uibk.ac.at’. All 25 sequencing runs were base-called using the highest-accuracy “SUP” model included in guppy using the flags “–num_callers 16 –gpu_runners_per_device 32 -x ‘auto’”. All reads that passed base calling were concatenated to a single file. This file was analysed using NanoPlot and PycoQC.

### 4.7. Genome Assembly

All reads that passed the base caller’s internal quality standards (Q-Score > 10; equals to a 10% error rate) were fed into flye v2.8.3 to be assembled into a draft genome with the following flags: ‘–nano-raw $input –threads 63 -g 400m -m 4000’. This draft genome was corrected using medaka v1.4.4 using the model ‘r941_min_sup_g507’ and the initial long reads that were used during assembly. The resulting polished draft genome was further polished using short 150 bp Illumina reads. Here, the short reads were aligned to the draft genome using bwa-mem 0.7.17-r1188, the result file was sorted using samtools v1.7, and pilon v1.24 (in a miniconda3 environment) was run with the flags ‘–frags $mapped_rnaseq_reads –changes –fix snps, indels’ to correct single-nucleotide polymorphisms (SNPs), as well as inserts and deletions (indels). Pilon polishing was performed in four subsequent iterations. Then, we used Purge Haplotigs v1.1.2 to remove duplicated contigs from the polished draft genomes using the following settings: “-l 5 -m 22 -h 120”. The mitogenome was salvaged from the pre-purged version of the genome (contig_79699), and the single contig was appended to the final genome.

#### Masking Repeats in the Genome

We used RepeatModeler v2.0.2 (with TRF v4.09, RECON, RepeatScout v1.0.6, and RepeatMasker v4.1.2) to detect repeat families in the polished draft genome with the flag “-LTRStruct” [58]. These repeat families were then fed into RepeatMasker (using NBCI/RMBlast v2.10.0+) with the optional flag to soft mask the genome “-xsmall”.

### 4.8. Protein Prediction and Genome Annotation

Protein prediction was performed using the braker2 v2.1.6 pipeline, with the *de novo* transcriptome as intrinsic information and the final polished and soft-masked genome as the template [59]. The software was run with the flags “–gff3 –softmasked”.

### 4.9. Quality Assessment of Transcriptome, Genome, and Protein Prediction

FastQC v0.11.9 was used to collect metrics about Illumina sequencing runs. NanoPlot v1.30.1 was used to assess the quality of the next-generation sequencing runs [60]. Quast v5.0.2 was used to collect metrics of the genome [61]. BUSCO v5.2.2 with the metazoa_odb10 dataset was used to check for completeness of the transcriptome, the genome, and the protein prediction [62,63].

### 4.10. Computational Integration of Sequencing Data

The assembled transcriptome and genome, as well as the predicted proteins, were added into a custom SequenceServer v2.0.0.rc4 instance [64] using BLASTN 2.10.0+. In addition, the genome was visualised in a Jbrowse server v1.16.9 with custom tracks: mapped transcriptomes, raw Illumina files (transcriptome RNA-seq files, head-specific RNA-seq files, tail-specific RNA-seq files, and gDNA files), repeatmasker files, and the Nanopore ultra-long reads. Both applications were hosted on a local Linux workstation.

### 4.11. Differential RNA-seq

The *de novo* transcriptome was further processed for downstream analyses using the transcript clustering tool cd-hit-est [65], which was invoked using the flags ‘-c 0.95 -d 0’. This software maps each transcript of the transcriptome to one another and adds them to one cluster if the identity is above or equal to 95%, keeping only the longest isoform from each cluster. The rationale behind the application of cd-hit-est is to reduce the complexity of the transcriptome to avoid the dilution of mapped reads to highly similar transcripts, thereby fostering the identification of differentially expressed genes. A total of 381,272 clusters were retained in the final transcriptome used for differential RNA-seq analysis. This transcriptome was indexed with salmon v1.4.0 [66], and all three biological replicates (single end 50 base pairs) of “heads” and “tails” were semi-quantified using salmon with the flags ‘-l A –validateMappings’. Then, the columns of the output file were switched, and the file headers ‘TXNAME’ and ‘GENEID’ were prepended. To receive the tx2gene.csv file, we invoked the support script get_Trinity_gene_to_trans_map.pl provided with Trinity v2.10.0. A sample text file was created containing information for the assay (head or tail), run (head_* or tail_* with the biological replicate 1–3), and the destination of the quantification file. Next, R v3.6.3 was started, and the libraries tximport, readr, tximportData, and DESeq2 were loaded. DESeq2 v1.26.0 was run on the dataset (using design = ~ assay) [67]. The final result file was exported as a column-separated values file.

### 4.12. Tail-Specific Candidate Genes Selection

We selected the 51 transcripts that showed the highest expression exclusively in the tail. The expression level of these transcripts ranged between a log_2_-fold change of 13.74 (>13.700 fold) and 5,64 (>50 fold). Each candidate transcript was BLASTed to the genome, and the matching contig regions were compared between transcripts to determine whether transcripts could potentially form part of a larger gene that was degraded into shorter, non-overlapping fragments in the transcriptome and thus mapped next to each other on the genomic contig.

### 4.13. Conserved Domain Search

We used the NCBI Conserved Domain database (CDD v3.19) with default settings to find conserved domains within the candidate genes [68]. In addition, we used signalP v6.0 and TmHMM v2.0 to predict a signal peptide and a transmembrane helix–loop–helix structure in the predicted proteins [69,70].

### 4.14. In Situ Hybridisations

#### 4.14.1. Probe Synthesis

For in situ hybridisation, digoxigenin (DIG)-labelled RNA probes were synthesised using a RNA DIG labelling (SP6/T7) kit (Roche, Basel, Switzerland) following the manufacturer’s instructions. In short, 6.5 µL of the cleaned-up template PCR product (primer sequences can be retrieved in the list “Theama_primerlist.xlsx” in doi:10.5281/zenodo.6470295) with a 5’-T7 and a 3’-SP6 overhang (or without this overhang but cloned into the pGEM-T vector) was combined with 1 µL of the labelling mix, 1 µL of the 10× transcription buffer, 0.5 µL RNAse inhibitor, and 1 µL RNA polymerase (T7 for sense control probes, SP6 for antisense in situ probes). The reaction was incubated for 2 h at 37 °C; then, 1 µL of DNAse I was added, mixed by agitation, and incubated for another 15 min at 37 °C. Then, 15 µL of nuclease-free water was added, and the total volume of 26 µL was purified using Micro Bio-Spin™ P-6 gel columns in SSC buffer (Bio-Rad, Hercules, CA, USA) according to the manufacturer’s protocol. The concentration of the DIG-labelled RNA probe was measured using NanoDrop, and the quality was verified on a 1% agarose gel (60 min, 120 V). The probes were diluted to 5 ng/µL in HybMix (50% formamide, 5 × SSC, 100 μg/mL heparin, 0.1% TWEEN^®^ 20 (Sigma-Aldrich, St. Louis, MO, USA), 0.1% CHAPS, 200 μg/mL yeast tRNA, 1 × Denhardt’s) prior to storage at −80 °C.

#### 4.14.2. Fixation for ISH

For in situ hybridisations (ISH), the animals had to be individually fixed and then treated in batches with a mucus-stripping solution to deprive them of their surrounding mucus, which heavily interferes during the development stage of the ISH. First, animals were incubated in the mucusstripping solution, consisting of 100 mg N-acetyl-L-cysteine (NAC) in 9 mL ASW, 350 µL HEPES-NaOH (pH 7.4), 5 µL phenol red, and 630 µL 1 M NaOH in a 15 mL tube [71]. The animals were kept on a platform rocker for exactly 10 min, immediately washed twice with ASW, and then individually relaxed. To this end, they were individually placed in a small drop of ASW onto a glass dish and were relaxed immediately by adding 7.14% MgCl_2_ × 6 H_2_O behind the animal and sucking it away immediately in front of the animal. The tendency of the animal to crawl into the shape of a ball was therefore avoided, remaining in an elongated form. After 15 s of relaxation, the animals were fixed with 4% formaldehyde (made from paraformaldehyde, PFA) in 0.1 M phosphate-buffered saline (*w*/*v*) in the same manner as described above. Animals were then fixed for 60 min in PFA, rinsed several times in 0.1 M PBS with 0.1% TWEEN^®^ 20, and dehydrated in an ascending (25%, 50%, 75%, 100%) methanol series in PBS series. After changing the 100% MeOH several times, the animals were stored at −20 °C.

#### 4.14.3. Whole Mount In Situ Hybridisation

In situ hybridisation was carried out as previously described in [72], with the following modifications: (1) rehydration was performed with methanol instead of ethanol; (2) proteinase K and heat fixation were changed to 15 min and 30 min, respectively; (3) the change from 100% HybMix to 2 × SSC was applied gradually (100% HybMix, 75% HybMix/25% 2 × SSC, 50% HybMix/50% 2 × SSC, 25% HybMix/75% 2 × SSC); (4) the labelled probe was used at a final concentration of 0.2 ng/µL in a volume of 200 µL; (5) hybridisation was performed overnight; and (6) the animals were mounted in Mowiol or in Aqua-Poly/Mount (Polysciences, Warrington, PA, USA).

### 4.15. RNA Interference

#### 4.15.1. Double-Stranded RNA Synthesis

Double-stranded RNA was synthesised by producing the sense and antisense strand in two different reactions. The RNA sense and antisense strands were produced using template PCR products for each gene of interest with a T7 (on 5’-end) and SP6 (on 3’-end) overhang and HiScribe™ T7 and SP6 RNA synthesis kits (New England Biolabs, Ipswich, MA, USA), following the manufacturer’s protocol (primer sequences can be retrieved in the list “Theama_primerlist.xlsx” in doi:10.5281/zenodo.6470295). Each of the reactions were incubated for 4 h at 37 °C in a thermocycler. Then, the sense and antisense reactions were mixed together to a final volume of 45 µL, and RNA was denatured for 5 min at 70 °C in a thermocycler. Then, the RNA mixture was gradually cooled down to room temperature by wrapping it in several layers of aluminium foil. Next, 2 µL of 0.5 mg/mL RNAse A was added to degrade leftover single-strand RNA, and 2 µL DNAse (Roche, Basel, Switzerland) was added to degrade template DNA strands. Degradation was performed for 30 min at 37 °C in a thermocycler. Alcohol precipitation was performed using 4.9 µL 3 M sodium acetate and 49 µL isopropanol. The reaction was immediately mixed by inversion of the tube and incubated for 5 min on ice until the reaction became cloudy. Then, the mixture was centrifuged for 30 min at 21.000× *g* in a pre-cooled (4 °C) centrifuge. The supernatant was completely removed, and the pellet was dislodged using 500 µL pre-cooled 75% ethanol in nuclease-free water prior to centrifugation at 7400× *g* for 5 min at 4 °C. The supernatant was subsequently completely removed and the pellet was air-dried for 10 min and then resuspended in 100 µL nuclease-free water. Double-stranded RNA was quality-checked with a 1% agarose gel (60 min, 120 V), aliquoted to 6 µL dsRNA samples, and immediately stored at −80 °C.

#### 4.15.2. dsRNA Treatment and Evaluation

For each treatment, the tails of 15 adult animals were amputated using a razor blade under a binocular without anaesthetisation. Then, all 15 animals were transferred into a glass embryo dish in 600 µL ASW. For each experiment, the dsRNA soaking solution was prepared containing 600 µL artificial sea water, 6 µL of the respective dsRNA, and 2 µL of antibiotics. The antibiotic was alternated every day between ampicillin (50 mg/mL), kanamycin (50 mg/mL), and streptomycin (10 mg/mL). Two different negative controls were run in parallel to the experiment: a control where no dsRNA was added, as well as an off-target control where dsRNA for a gene that is not present in flatworms, namely *Luciferase* (pGEM-luc Vector, Promega, Madison, WI, USA), was added to the worms. Every day, the medium was completely removed, and the animals were washed twice with ASW and immediately soaked again in a freshly prepared dsRNA soaking mixture. We checked daily for adhesion over a total of 14 days. Three researchers performed a blind test on three individuals for each experiment, resulting in a total of nine observed animals per experiment.

### 4.16. Lectin Stainings

For lectin stainings, animals were fixed in the same manner as described in the Section 4.14. and stored in 100% methanol at −20 °C. To rehydrate them, animals were incubated for 5 min each in a descending methanol series (100%, 75%, 50%, 25%) with 1 × Tris based-saline with 0.01% Triton™ X-100 (Sigma-Aldrich, St. Louis, MO, USA) (TBS-T_x_) and 5 mM CaCl_2_. Next, animals were washed several times in TBS-T_x_ (as above) prior to being blocked overnight in TBS-T_x_ with 3% (*w*/*v*) bovine serum albumin (TBS-T_x_-BSA). Next, the animals were incubated for 2 h at room temperature in 25 µg/mL lectin in TBS-T_x_-BSA. Then, animals were washed several times for 1 h to remove the unbound lectins with TBS-T_x_. Then, Texas Red Streptavidin (Vector Laboratories, Burlingame, CA, USA) was diluted 1:500, added to the samples, and incubated for 2 h in darkness at room temperature prior to being washed several times over the course of one hour in darkness at room temperature. Samples were then mounted in VectaShield^®^ antifade medium (Vector Laboratories, Burlingame, CA, USA) on glass slides. The slides were then stored at −20 °C.

### 4.17. L-DOPA Staining

Fluorescent antibody staining was performed on footprints of *T. mediterranea*. To obtain footprints, 5 animals were kept in a small droplet of ASW on a microscope slide with discontinuous movement by pipetting the ASW to induce the animals to attach themselves to the glass slide. Then, footprints were fixed for 30 min in 4% PFA. After washing for 1 h in TBS-T_x_ + 5 mM CaCl_2_, they were incubated in TBS-T_x_-BSA for 1 h at room temperature and then incubated in a mixture of 1:500 rabbit-α-DOPA (ab6426, Abcam, Cambridge, UK) and 20 µg/mL biotinylated peanut agglutinin lectin for 1 h at room temperature. Then, they were washed several times in TBS-T_x_ + 5 mM CaCl_2_ over the course of 1 h at room temperature. Next, they were incubated in a mixture of the secondary antibody swine-α-rabbit FITC (F0205, Dako/Agilent, Santa Clara, CA, USA) 1:500 and Texas Red Streptavidin (Vector Laboratories, Burlingame, CA, USA) diluted to 1:500 for one hour at room temperature in darkness. Then, they were washed several times during the course of one hour in darkness at room temperature prior to being mounted (see chapter on lectin stainings). In the negative controls, the L-DOPA antibody was omitted. In addition, three different secondary antibodies against rabbit antibodies were tested: goat-a-rabbit Alexa Fluor 488 (A31627A, Invitrogen, Waltham, MA, USA), goat-α-rabbit Alexa Fluor 568 (A11036, Invitrogen, Waltham, MA, USA), and goat-α-rabbit TRITC (111-025-144, Jackson Immuno Research Labs, Baltimore, PA, USA). Note that we switched to Alexa Fluor 488 Streptavidin (Vector Laboratories, Burlingame, CA, USA) when using goat-α-rabbit Alexa Fluor 568 or goat-α-rabbit TRITC.

Nitroblue tetrazolium staining solution was prepared as described by Zeng et al. [6]. Footprints were fixed for 1 h at RT in 4% PFA in PBS, washed for 1 h with 1 × PBS-T_x_, and then stained for 20 min at RT. Then, the footprints were washed for 30 min in PBS-T_x_ prior to being mounted in Aqua-Poly/Mount (Polysciences, Warrington, PA, USA).

### 4.18. Fixation for Transmission Electron Microscopy

Electron microscopy was carried out according to Wunderer et al. [26].

## Figures and Tables

**Table 1:** Characteristics of the adhesive proteins and potential adhesion-related proteins in *T. mediterranea*. The length of the protein is given in amino acid residues. The presence of a signal peptide (as predicted by signalP v6.0) or a transmembrane domain (TMHMM v2.0) is shown, as well as the predicted molecular weight in kilo Dalton and the predicted isoelectric point (IP).

Protein Name	Accession	aa	sigP	TMM	Weight (kDa)	IP	Comments
Tmed-ap1	ON323669	1687	yes	no	187.484	6.28	Named after [26]
Tmed-ap2	ON323670	7228	yes	no	797.113	10.47	Named after [26]
Tmed-ap3	ON323671	3700	yes	no	441.653	13.24	Named after [28]
Tmed-7752	ON323672	308	yes	no	34.197	9.6	
Tmed-tyr1	ON323673	511	yes	no	60.67	11.01	Secreted tyrosinase
Tmed-9797	ON323674	85	yes	no	9.705	7.62	
Tmed-10419	ON323675	141	no	yes	15.724	8.96	Predicted to have a transmembrane domain
Tmed-capeuk	ON323676	308	yes	no	33.24	7.54	Contains cysteine-rich secretory protein domain
Tmed-21993	ON323677	130	yes	no	14.134	8.81	
Tmed-ctl1	ON323678	210	yes	no	23.903	8.79	Contains a c-type lectin binding domain
Tmed-if1	ON323679	671	no	no	76.258	6.16	Named after [29]
Tmed-krg1	ON323680	1357	no	yes	145.787	4.87	Contains six Kringle domains
Tmed-14707	ON323681	131	yes	no	16.152	8.67	19.1% arginine
Tmed-66071	ON323682	89	no	no	10.286	6.36	
Tmed-51251	ON323683	573	no	no	66.881	5.26	

**Table 2:** Overview of high-molecular-weight genomic DNA extraction methods. Abbreviations: NAC, N-acetyl-L-cysteine in mucus removal solution (see Section 4.14); SRE, short-read eliminator kit. The accession numbers are deposited in the NCBI BioSample database.

	Mucus Removal	CT-Buffer	SRE	Runs	Accession
Library 1	100 mg NAC	yes	-	1	SAMN27735442
Library 2	100 mg NAC	yes	-	1	SAMN27735443
Library 3	50 mg NAC	yes	XS + normal	4	SAMN27735444
Library 4	100 mg NAC	yes	XS + normal	3	SAMN27735445
Library 5	no	no	XS + normal	2	SAMN27735446
Library 6	50 mg NAC	yes	XS + normal	3	SAMN27735447
Library 7	50 mg NAC	yes	XS + normal	4	SAMN27735448
Library 8	50 mg NAC	yes	XL	4	SAMN27735449
Library 9	no NAC	yes	XL	3	SAMN27735450

## Data Availability

The sequences shown in this work are deposited in NCBI under the accession numbers Tmed-ap1 (ON323669), Tmed-ap2 (ON323670), Tmed-ap3 (ON323671), Tmed-7752 (ON323672), Tmed-tyr1 (ON323673), Tmed-9797 (ON323674), Tmed-10419 (ON323675), Tmed-capeuk (ON323676), Tmed-21993 (ON323677), Tmed-ctl1 (ON323678), Tmed-if1 (ON323679), Tmed-krg1 (ON323680), Tmed-14707 (ON323681), Tmed-66071 (ON323682), and Tmed-51251 (ON323683). The native Nanopore sequencing files of all nine libraries are deposited at NCBI BioSamples SAMN27735442, SAMN27735443, SAMN27735444, SAMN27735445, SAMN27735446, SAMN27735447, SAMN27735448, SAMN27735449, SAMN27735450. Genomic and transcriptomic Illumina reads (RNA-seq, RNA-diff, gDNA) as well as the annotated transcriptome, the genome, all annotation files, and the differential RNA-seq analysis data, are deposited in the Zenodo database accessible at https://doi.org/10.5281/zenodo.6470295 (accessed on 30 April 2022), Version 1.

## Supplementary Materials

The following supporting information can be downloaded at: https://www.mdpi.com/article/10.3390/md20060359/s1, Figure S1: Details of adhesive field; Figure S2: Different transcripts mapped to adhesion protein 3; Figure S3: Sense-control in situ hybridisations of the 15 candidate genes; Figure S4: Peanut agglutinin lectin staining in animals and their footprints; Figure S5: Protein content shown by nitrogen distribution in the adhesive vesicles of *T. mediterranea*; Table S1: Metrics of the four RNA paired end 150 base pair reads sequencing runs; Table S2: Metrics of the six sequencing results of the differential RNA-seq analysis.

## References

[1] Bianco-Peled H., Davidovich-Pinhas M. (2015). Bioadhesion and Biomimetics: From Nature to Applications.

[2] Smith A.M. (2016). Biological Adhesives.

[3] von Byern J., Grunwald I. (2010). Biological Adhesive Systems: From Nature to Technical and Medical Application.

[4] Lengerer B., Ladurner P. (2018). Properties of Temporary Adhesion Systems of Marine and Freshwater Organisms. J. Exp. Biol..

[5] Whittington I.D., Cribb B.W. (2001). Adhesive Secretions in the Platyhelminthes. Adv. Parasitol..

[6] Zeng F., Wunderer J., Salvenmoser W., Ederth T., Rothbächer U. (2019). Identifying Adhesive Components in a Model Tunicate. Philos. Trans. R. Soc. B.

[7] Lu Q., Danner E., Waite J.H., Israelachvili J.N., Zeng H., Hwang D.S. (2013). Adhesion of Mussel Foot Proteins to Different Substrate Surfaces. J. R. Soc. Interface.

[8] Waite J.H. (2017). Mussel Adhesion—Essential Footwork. J. Exp. Biol..

[9] Lee H., Scherer N.F., Messersmith P.B. (2006). Single-Molecule Mechanics of Mussel Adhesion. Proc. Natl. Acad. Sci. USA.

[10] Waite J.H., Tanzer M.L. (1981). Polyphenolic Substance of *Mytilus edulis*: Novel Adhesive Containing L-Dopa and Hydroxyproline. Science.

[11] Davey P.A., Power A.M., Santos R., Bertemes P., Ladurner P., Palmowski P., Clarke J., Flammang P., Lengerer B., Hennebert E. (2021). Omics-Based Molecular Analyses of Adhesion by Aquatic Invertebrates. Biol. Rev..

[12] Flammang P., Colliec-Jouault S., Bergé J.P. (2003). The glue of sea cucumber Cuvierian tubules: A novel marine bioadhesive. Marine Biotechnology: An Overview of Leading Fields.

[13] Gaspar L., Flammang P., José R., Luis R., Ramalhosa P., Monteiro J., Nogueira N., Canning-Clode J., Santos R. (2021). Interspecific Analysis of Sea Urchin Adhesive Composition Emphasizes Variability of Glycans Conjugated with Putative Adhesive Proteins. Front. Mar. Sci..

[14] Hennebert E., Wattiez R., Waite J.H., Flammang P. (2012). Characterization of the Protein Fraction of the Temporary Adhesive Secreted by the Tube Feet of the Sea Star *Asterias rubens*. Biofouling.

[15] Hennebert E., Wattiez R., Demeuldre M., Ladurner P., Hwang D.S., Waite J.H., Flammang P. (2014). Sea Star Tenacity Mediated by a Protein That Fragments, Then Aggregates. Proc. Natl. Acad. Sci. USA.

[16] Lengerer B., Bonneel M., Lefevre M., Hennebert E., Leclère P., Gosselin E., Ladurner P., Flammang P. (2018). The Structural and Chemical Basis of Temporary Adhesion in the Sea Star *Asterina gibbosa*. Beilstein J. Nanotechnol..

[17] Lengerer B., Algrain M., Lefevre M., Delroisse J., Hennebert E., Flammang P. (2019). Interspecies Comparison of Sea Star Adhesive Proteins. Philos. Trans. R. Soc. B Biol. Sci..

[18] Pjeta R., Lindner H., Kremser L., Salvenmoser W., Sobral D., Ladurner P., Santos R. (2020). Integrative Transcriptome and Proteome Analysis of the Tube Foot and Adhesive Secretions of the Sea Urchin *Paracentrotus lividus*. Int. J. Mol. Sci..

[19] Santos R., Barreto A., Franco C., Coelho A.V. (2013). Mapping Sea Urchins Tube Feet Proteome—A Unique Hydraulic Mechano-Sensory Adhesive Organ. J. Proteomics.

[20] Santos R., Flammang P. (2012). Is the Adhesive Material Secreted by Sea Urchin Tube Feet Species-Specific?. J. Morphol..

[21] Hennebert E., Vivilleb P., Lazzaroni R., Flammang P. (2008). Micro- and Nanostructure of the Adhesive Material Secreted by the Tube Feet of the Sea Star Asterias rubens. J. Struct. Biol..

[22] Hennebert E., Wattiez R., Flammang P. (2011). Characterisation of the Carbohydrate Fraction of the Temporary Adhesive Secreted by the Tube Feet of the Sea Star *Asterias rubens*. Mar. Biotechnol..

[23] Tyler S. (1976). Comparative Ultrastructure of Adhesive Systems in the Turbellaria. Zoomorphologie.

[24] Egger B., Lapraz F., Tomiczek B., Müller S., Dessimoz C., Girstmair J., Škunca N., Rawlinson K.A., Cameron C.B., Beli E. (2015). A Transcriptomic-Phylogenomic Analysis of the Evolutionary Relationships of Flatworms. Curr. Biol..

[25] Laumer C.E., Hejnol A., Giribet G. (2015). Nuclear Genomic Signals of the ‘Microturbellarian’ Roots of Platyhelminth Evolutionary Innovation. eLife.

[26] Wunderer J., Lengerer B., Pjeta R., Bertemes P., Kremser L., Lindner H., Ederth T., Hess M.W., Stock D., Salvenmoser W. (2019). A Mechanism for Temporary Bioadhesion. Proc. Natl. Acad. Sci. USA.

[27] Bertemes P., Pjeta R., Wunderer J., Grosbusch A.L., Lengerer B., Grüner K., Knapp M., Mertens B., Andresen N., Hess M.W. (2021). (Un)Expected Similarity of the Temporary Adhesive Systems of Marine, Brackish, and Freshwater Flatworms. Int. J. Mol. Sci..

[28] Pjeta R., Wunderer J., Bertemes P., Hofer T., Salvenmoser W., Lengerer B., Coassin S., Erhart G., Beisel C., Sobral D. (2019). Temporary Adhesion of the Proseriate Flatworm *Minona ileanae*. Philos. Trans. R. Soc. B Biol. Sci..

[29] Lengerer B., Pjeta R., Wunderer J., Rodrigues M., Arbore R., Schärer L., Berezikov E., Hess M.W., Pfaller K., Egger B. (2014). Biological Adhesion of the Flatworm *Macrostomum lignano* Relies on a Duo-Gland System and Is Mediated by a Cell Type-Specific Intermediate Filament Protein. Front. Zool..

[30] Lengerer B., Hennebert E., Flammang P., Salvenmoser W., Ladurner P. (2016). Adhesive Organ Regeneration in *Macrostomum lignano*. BMC Dev. Biol..

[31] Bertemes P., Grosbusch A.L., Egger B. (2020). No Head Regeneration Here: Regeneration Capacity and Stem Cell Dynamics of *Theama mediterranea* (Polycladida, Platyhelminthes). Cell Tissue Res..

[32] Curini-Galletti M., Campus P., Delogu V. (2008). *Theama mediterranea* Sp. Nov. (Platyhelminthes, Polycladida), the First Interstitial Polyclad from the Mediterranean. Ital. J. Zool..

[33] Hennebert E., Leroy B., Wattiez R., Ladurner P. (2015). An Integrated Transcriptomic and Proteomic Analysis of Sea Star Epidermal Secretions Identifies Proteins Involved in Defense and Adhesion. J. Proteom..

[34] Kang V., Lengerer B., Wattiez R., Flammang P. (2019). Molecular Insights into the Powerful Mucus-Based Adhesion of Limpets (*Patella vulgata* L.). Open Biol..

[35] Ohkawa K., Nishida A., Yamamoto H., Waite J.H. (2004). A Glycosylated Byssal Precursor Protein from the Green Mussel *Perna viridis* with Modified Dopa Side-Chains. Biofouling.

[36] Pagett H.E., Abrahams J.L., Bones J., O’Donoghue N., Marles-Wright J., Lewis R.J., Harris J.R., Caldwell G.S., Rudd P.M., Clare A.S. (2012). Structural Characterisation of the N-Glycan Moiety of the Barnacle Settlement-Inducing Protein Complex (SIPC). J. Exp. Biol..

[37] Simão M., Moço M., Marques L., Santos R. (2020). Characterization of the Glycans Involved in Sea Urchin *Paracentrotus lividus* Reversible Adhesion. Mar. Biol..

[38] Flammang P., Lambert A., Bailly P., Hennebert E. (2009). Polyphosphoprotein-Containing Marine Adhesives. J. Adhes..

[39] Stewart R.J., Weaver J.C., Morse D.E., Waite J.H. (2004). The Tube Cement of *Phragmatopoma californica*: A Solid Foam. J. Exp. Biol..

[40] Hennebert E., Gregorowicz E., Flammang P. (2018). Involvement of Sulfated Biopolymers in Adhesive Secretions Produced by Marine Invertebrates. Biol. Open.

[41] Wang C.S., Stewart R.J. (2012). Localization of the Bioadhesive Precursors of the Sandcastle Worm, *Phragmatopoma californica* (Fewkes). J. Exp. Biol..

[42] Öztürk E., Stauber T., Levinson C., Cavalli E., Arlov Ø., Zenobi-Wong M. (2020). Tyrosinase-Crosslinked, Tissue Adhesive and Biomimetic Alginate Sulfate Hydrogels for Cartilage Repair. Biomed. Mater..

[43] Ryu J.H., Hong S., Lee H. (2015). Bio-Inspired Adhesive Catechol-Conjugated Chitosan for Biomedical Applications: A Mini Review. Acta Biomater..

[44] Nicklisch S., Waite J.H. (2012). Mini-Review: The Role of Redox in DOPA-Mediated Marine Adhesion. Biofouling.

[45] Davey P.A., Rodrigues M., Clarke J.L., Aldred N. (2019). Transcriptional Characterisation of the *Exaiptasia pallida* Pedal Disc. BMC Genom..

[46] Choi S., Ahn H., Kim S.-H. (2022). Tyrosinase-Mediated Hydrogel Crosslinking for Tissue Engineering. J. Appl. Polym. Sci..

[47] Kim S.-H., Lee S.-H., Lee J.-E., Park S.J., Kim K., Kim I.S., Lee Y.-S., Hwang N.S., Kim B.-G. (2018). Tissue Adhesive, Rapid Forming, and Sprayable ECM Hydrogel via Recombinant Tyrosinase Crosslinking. Biomaterials.

[48] Tulinsky A., Park C.H., Mao B., Llináas M. (1988). Lysine/fibrin binding sites of kringles modeled after the structure of kringle 1 of prothrombin. Proteins Struct. Funct. Bioinform..

[49] Castellino F.J., McCance S.G. (1997). The Kringle Domains of Human Plasminogen. Plasminogen-Relat. Growth Factors.

[50] Vlahos C.J., Wilhelm O.G., Hassell T., Jaskunas S.R., Bang N.U. (1991). Disulfide Pairing of the Recombinant Kringle-2 Domain of Tissue Plasminogen Activator Produced in *Escherichia coli*. J. Biol. Chem..

[51] Mizuno K., Inoue H., Hagiya M., Shimizu S., Nose T., Shimohigashi Y., Nakamura T. (1994). Hairpin Loop and Second Kringle Domain Are Essential Sites for Heparin Binding and Biological Activity of Hepatocyte Growth Factor. J. Biol. Chem..

[52] Sigurdardottir A., Winter A., Sobkowicz A., Fragai M., Chirgadze D., Ascher B.D., Blundell L.T., Gherardi E. (2015). Exploring the Chemical Space of the Lysine-Binding Pocket of the First Kringle Domain of Hepatocyte Growth Factor/Scatter Factor (HGF/SF) Yields a New Class of Inhibitors of HGF/SF-MET Binding. Chem. Sci..

[53] Arbore R., Sekii K., Beisel C., Ladurner P., Berezikov E., Scharer L. (2015). Positional RNA-Seq Identifies Candidate Genes for Phenotypic Engineering of Sexual Traits. Front. Zool..

[54] Lengerer B., Wunderer J., Pjeta R., Carta G., Kao D., Aboobaker A., Beisel C., Berezikov E., Salvenmoser W., Ladurner P. (2018). Organ Specific Gene Expression in the Regenerating Tail of *Macrostomum lignano*. Dev. Biol..

[55] Song L., Florea L. (2015). Rcorrector: Efficient and Accurate Error Correction for Illumina RNA-Seq Reads. GigaScience.

[56] Krueger F., James F., Ewels P., Ebrahim Afyounian B.S.-B. TrimGalore: V0.6.7. 2021.

[57] Grabherr M.G., Haas B.J., Yassour M., Levin J.Z., Thompson D.A., Amit I., Adiconis X., Fan L., Raychowdhury R., Zeng Q. (2011). Full-Length Transcriptome Assembly from RNA-Seq Data without a Reference Genome. Nat. Biotechnol..

[58] Flynn J.M., Hubley R., Goubert C., Rosen J., Clark A.G., Feschotte C., Smit A.F. (2020). RepeatModeler2 for Automated Genomic Discovery of Transposable Element Families. Proc. Natl. Acad. Sci. USA.

[59] Brůna T., Hoff K.J., Lomsadze A., Stanke M., Borodovsky M. (2021). BRAKER2: Automatic Eukaryotic Genome Annotation with GeneMark-EP+ and AUGUSTUS Supported by a Protein Database. NAR Genom. Bioinform..

[60] De Coster W., D’Hert S., Schultz D.T., Cruts M., Van Broeckhoven C. (2018). NanoPack: Visualizing and Processing Long-Read Sequencing Data. Bioinformatics.

[61] Gurevich A., Saveliev V., Vyahhi N., Tesler G. (2013). QUAST: Quality Assessment Tool for Genome Assemblies. Bioinforma. Oxf. Engl..

[62] Manni M., Berkeley M.R., Seppey M., Simão F.A., Zdobnov E.M. (2021). BUSCO Update: Novel and Streamlined Workflows along with Broader and Deeper Phylogenetic Coverage for Scoring of Eukaryotic, Prokaryotic, and Viral Genomes. Mol. Biol. Evol..

[63] Seppey M., Manni M., Zdobnov E.M. (2019). BUSCO: Assessing Genome Assembly and Annotation Completeness. Methods Mol. Biol..

[64] Priyam A., Woodcroft B.J., Rai V., Moghul I., Munagala A., Ter F., Chowdhary H., Pieniak I., Maynard L.J., Gibbins M.A. (2019). Sequenceserver: A Modern Graphical User Interface for Custom BLAST Databases. Mol. Biol. Evol..

[65] Fu L., Niu B., Zhu Z., Wu S., Li W. (2012). CD-HIT: Accelerated for Clustering the next-Generation Sequencing Data. Bioinformatics.

[66] Patro R., Duggal G., Love M.I., Irizarry R.A., Kingsford C. (2017). Salmon Provides Fast and Bias-Aware Quantification of Transcript Expression. Nat. Methods.

[67] Love M.I., Huber W., Anders S. (2014). Moderated Estimation of Fold Change and Dispersion for RNA-Seq Data with DESeq2. Genome Biol..

[68] Lu S., Wang J., Chitsaz F., Derbyshire M.K., Geer R.C., Gonzales N.R., Gwadz M., Hurwitz D.I., Marchler G.H., Song J.S. (2020). CDD/SPARCLE: The Conserved Domain Database in 2020. Nucleic Acids Res..

[69] Krogh A., Larsson B., von Heijne G., Sonnhammer E.L. (2001). Predicting Transmembrane Protein Topology with a Hidden Markov Model: Application to Complete Genomes. J. Mol. Biol..

[70] Teufel F., Almagro Armenteros J.J., Johansen A.R., Gíslason M.H., Pihl S.I., Tsirigos K.D., Winther O., Brunak S., von Heijne G., Nielsen H. (2022). SignalP 6.0 Predicts All Five Types of Signal Peptides Using Protein Language Models. Nat. Biotechnol..

[71] Grohme M.A., Vila-Farré M., Rink J.C., Rink J.C. (2018). Small- and Large-Scale High Molecular Weight Genomic DNA Extraction from Planarians. Planarian Regeneration: Methods and Protocols.

[72] Pfister D., De Mulder K., Philipp I., Kuales G., Hrouda M., Eichberger P., Borgonie G., Hartenstein V., Ladurner P. (2007). The Exceptional Stem Cell System of *Macrostomum lignano*: Screening for Gene Expression and Studying Cell Proliferation by Hydroxyurea Treatment and Irradiation. Front. Zool..

