# Supplementary material for: Sticking Together an Updated Model for Temporary Adhesion
Source: Mar Drugs. 2022 May 27;20(6):359. doi: 10.3390/md20060359 (PMC9229212; doi:10.3390/md20060359)
Supplement: Supplementary file 1 [file marinedrugs-20-00359-s001.zip › marinedrugs-1727382-supplementary.pdf]

SUPPLEMENTARY MATERIAL

Sticking together an updated model for temporary adhesion

Philip Bertemes, Alexandra L. Grosbusch, Anik Geschwindt, Bob Kauffmann, Willi Salvenmoser, Birte Mertens, Robert Pjeta, Bernhard Egger, and Peter Ladurner*

Author affiliations
Institute of Zoology, University of Innsbruck, 6020 Innsbruck, Austria and Center of Molecular Biosciences Innsbruck, University of Innsbruck, 6020 Innsbruck, Austria

*) corresponding author: peter.ladurner@uibk.ac.at

ORCIDs

Philip Bertemes	0000-0002-8892-1538
Alexandra L. Grosbusch	0000-0002-5439-6667
Anik Geschwindt	0000-0002-5191-9039
Bob Kauffmann	0000-0003-0836-2228
Willi Salvenmoser	0000-0003-3361-0326
Birte Mertens	0000-0002-3710-1863
Robert Pjeta	0000-0002-5389-6950
Bernhard Egger	0000-0001-6353-9458
Peter Ladurner	0000-0002-0323-9266

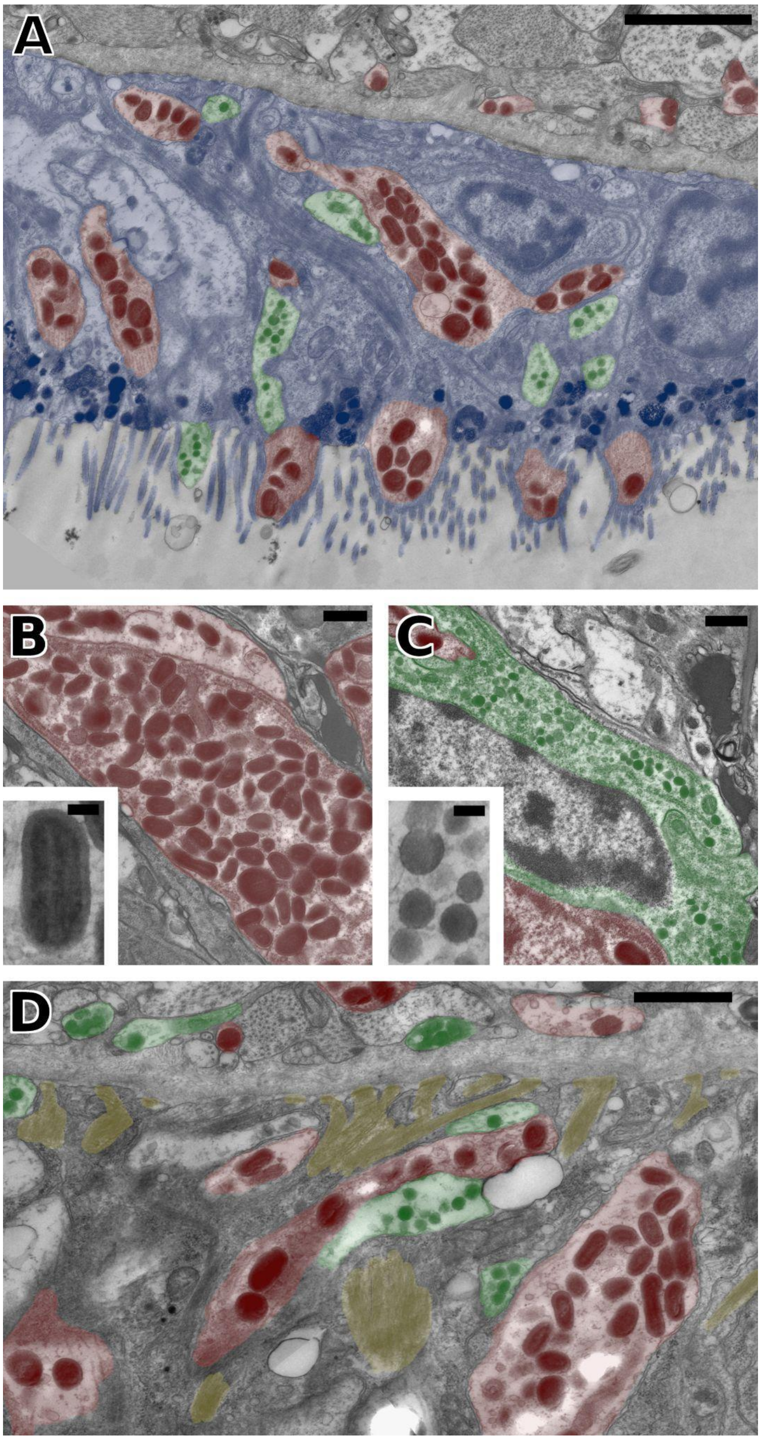

Supplementary Figure S1 Details of adhesive field.
A Overview of the adhesive field with the anchor cell (blue), the adhesive gland necks (red) and releasing gland necks (green). Detail of **B** one adhesive cell (inset: detail of adhesive vesicle with lucid outer ring, dense inner core, and substructures in the inner core) and **C** one releasing gland cell (inset: detail of releasing granules). **D** Intermediate filaments (yellow) attached with hemidesmosomes to the basal matrix. Scale bars: A: 2 µm, B, C: 500 nm; insets in B, C: 100 nm; D: 1 µm.

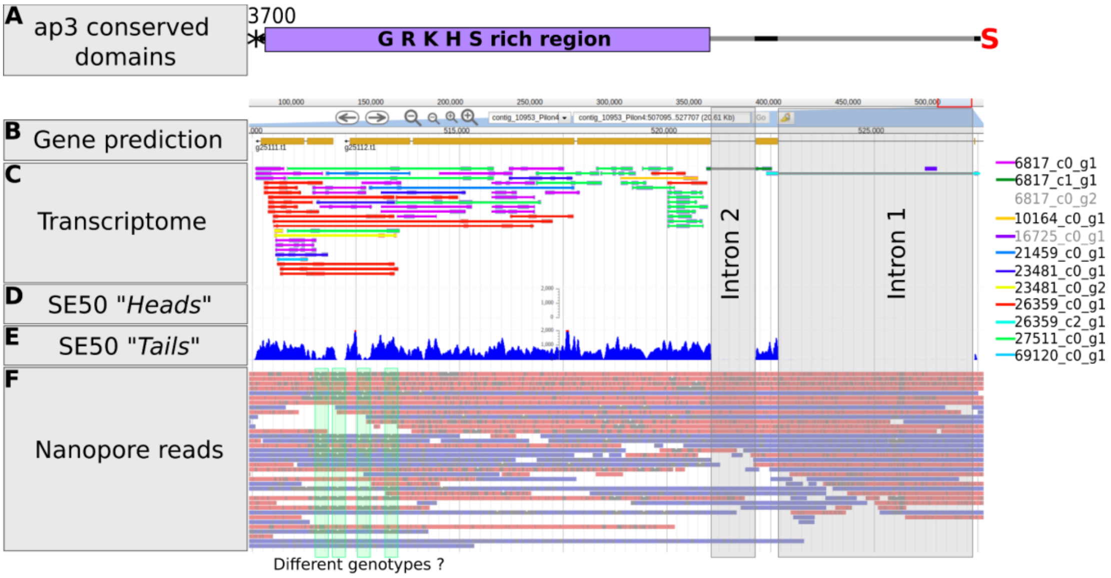

Supplementary Figure S2 Different transcripts mapped to adhesion protein 3.
A The gene architecture (S, signal peptide) of adhesion protein 3 (note that left is the C-terminal end, asterisk marks stop). **B** The gene prediction algorithm split ap3 into two different predictions, most probably due to frameshifts and/or an erroneously assembled region in the draft genome. **C** The transcripts that mapped to adhesion protein 3 gene region on the genome (different colours show different trinity “genes”. Each colour shows multiple trinity “isoforms” of the trinity “gene”). Number of mapped SE50 reads from the (**D**) “head” and the (**E**) “tail” region. Note that in the tail, read numbers up to 2496 single reads/base were reported. **F** The aligned long (Nanopore) reads onto this genomic region. Note the four green boxes, where reads with similar stretches of large deletions were reported.

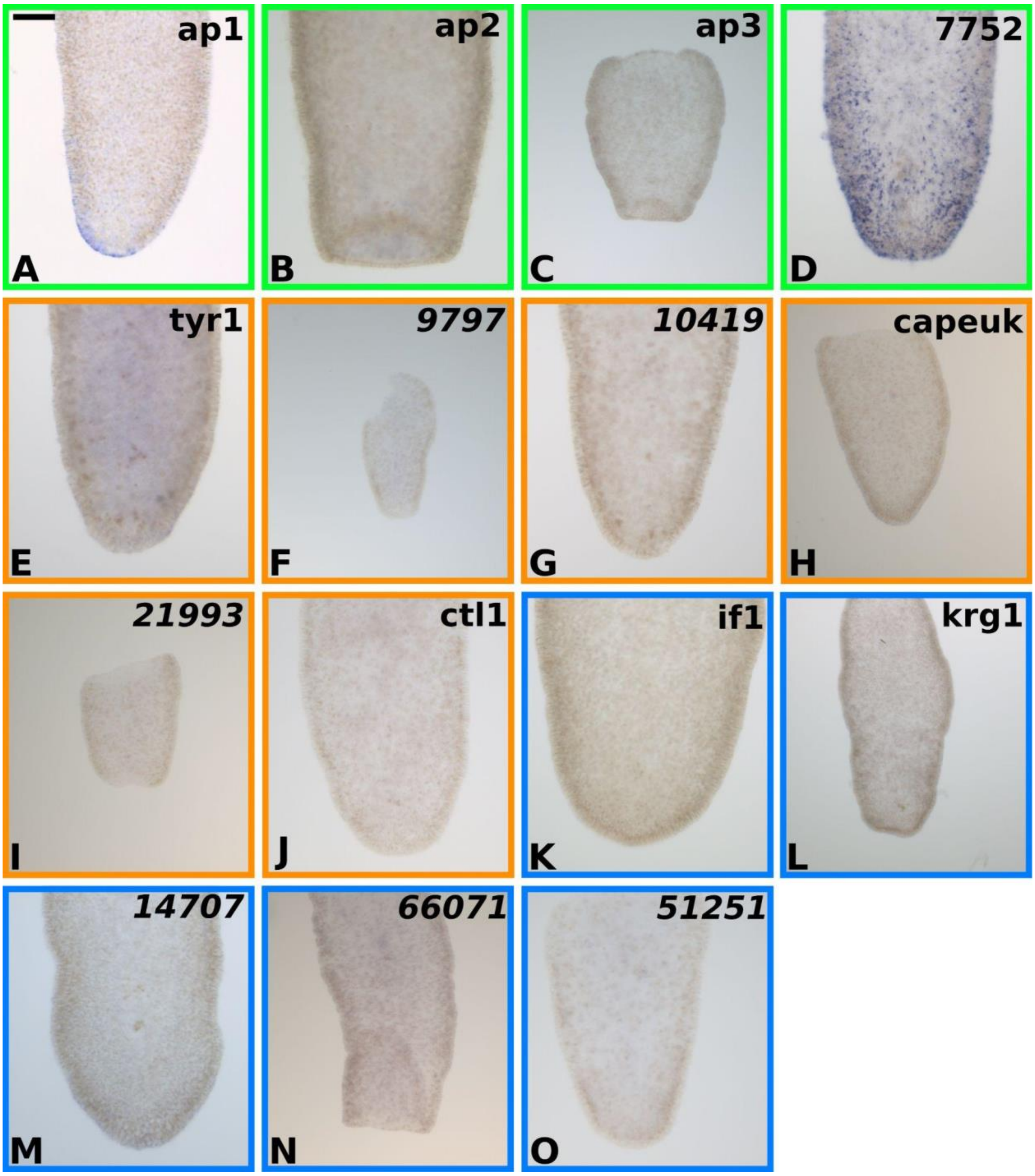

Supplementary Figure S3 Sense-control in situ hybridisations of the 15 candidate genes.
A-O sense-control *in situ* hybridisations for each tested gene. Note that the tails in **B**, **C**, and **N** are collapsed onto the body, but are still present. The sense-probe in **D** produces a high background, most probably it interacted with the rhabdites of the animal. Scale bar: 50 µm.

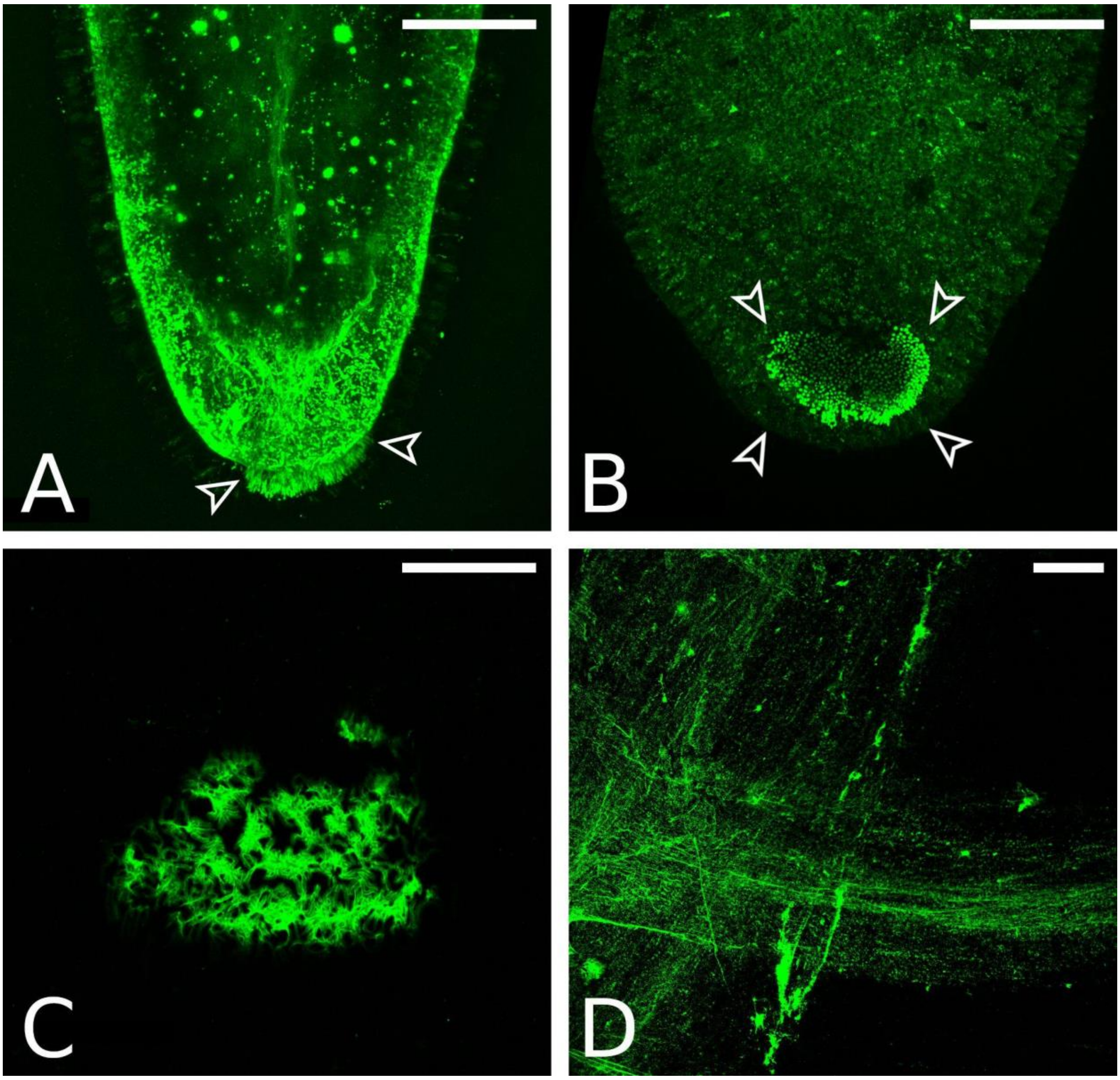

Supplementary Figure S4 Peanut agglutinin lectin staining in animals and their footprints.
A Whole mount adult animal, side view. Note how the adhesive gland necks run through the epidermal layer (area between the hollow arrow heads). **B** ventral view of the adhesive field with protruding adhesive gland cell necks. **C** PNA-positive footprint that was left behind by *T. mediterranea*. **D** PNA-positive slime produced and left behind on the glass slide of *T. mediterranea*. Scale bars: 50 µm (A-D).

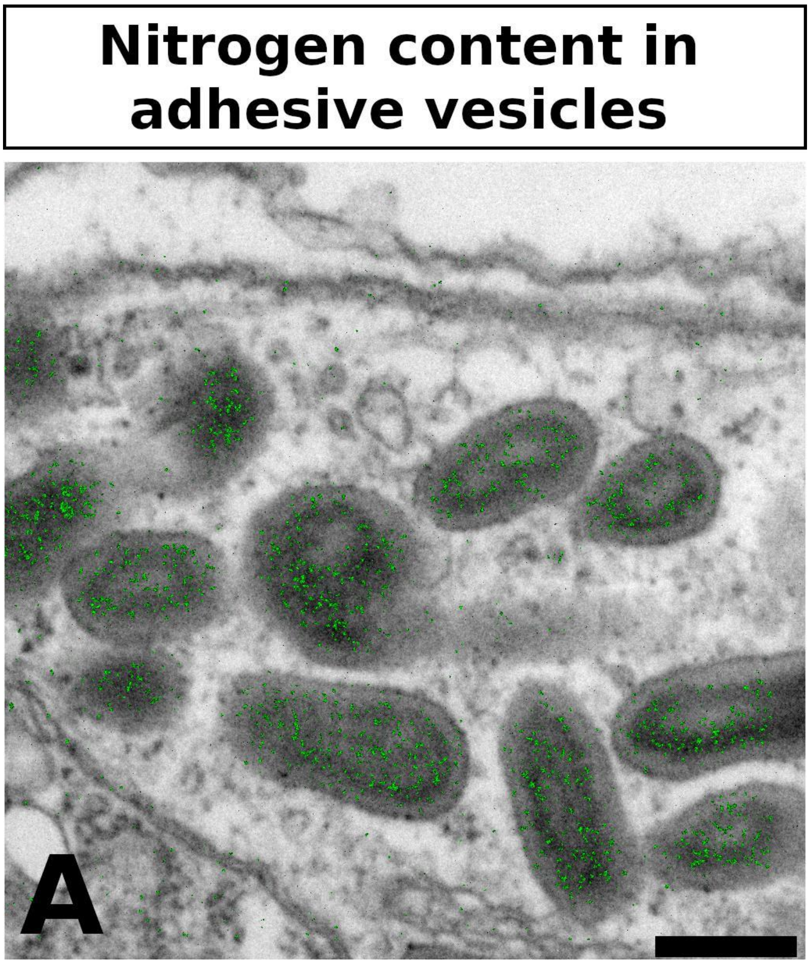

Supplementary Figure S5 Protein content shown by nitrogen distribution in the adhesive vesicles of *T. mediterranea*.

A A high number of nitrogen (green marks) was detected in the dark inner core of the adhesive vesicles compared to their more translucent outer rim with Electron Spectroscopic Imaging (ESI) on the transmission electron microscope. Scale bar: 200 nm.

Supplementary Table S1 Metrics of the four RNA paired end 150 base pair reads sequencing runs. First row shows the amount of raw reads, the second round the amount of base pairs that were changed by rcorrector and third line accounts for the amount of reads after rcorrector processing. Fourth and Fifth line show the amount of reads with adapters in reads R1 and R2, respectively. Sixth row shows the final reads for each replicate that were used for transcriptome assembly. Last row shows the GC content of the reads of each replicate.

	Replicate 1	Replicate 2	Replicate 3	Replicate 4
Raw reads	24,979,894	31,211,449	24,302,133	23,606,852
Corrected bases	28,807,843	43,927,039	31,094,981	29,808,230
Reads after rcorrector	20,616,823	27,406,306	19,979,327	19,051,150
Reads with adapters (R1)	9,396,347	10,376,625	9,006,928	9,347,469
Reads with adapters (R2)	9,664,380	11,020,451	9,176,435	9,437,278
Final reads	20,576,116	27,357,917	19,923,080	19,030,978
GC content [%]	44	43	44	44

Supplementary Table S2 Metrics of the six sequencing results of the differential RNA-seq analysis. Three biological replicates for animals where the tail was amputated (termed as “Head”) and the respective tails (termed as “Tail”).

	Head 1	Head 2	Head 3	Tail 1	Tail 2	Tail 3
reads	40,244,379	31,555,852	32,505,862	31,114,721	26,783,854	25,389,814
GC [%]	45	45	44	45	44	45
